# Massive Accumulation of Strontium and Barium in Diplonemid Protists

**DOI:** 10.1128/mbio.03279-22

**Published:** 2023-01-16

**Authors:** Jana Pilátová, Daria Tashyreva, Jiří Týč, Marie Vancová, Syed Nadeem Hussain Bokhari, Radim Skoupý, Mariana Klementová, Hendrik Küpper, Peter Mojzeš, Julius Lukeš

**Affiliations:** a Institute of Parasitology, Biology Centre, Czech Academy of Sciences, České Budějovice, Czech Republic; b Faculty of Science, Charles University, Prague, Czech Republic; c Institute of Physics, Faculty of Mathematics and Physics, Charles University, Prague, Czech Republic; d Faculty of Sciences, University of South Bohemia, České Budějovice, Czech Republic; e Institute of Plant Molecular Biology, Biology Centre, Czech Academy of Sciences, České Budějovice, Czech Republic; f Institute of Scientific Instruments, Czech Academy of Sciences, Brno, Czech Republic; g Institute of Physics, Czech Academy of Sciences, Prague, Czech Republic; Washington University School of Medicine

**Keywords:** Euglenozoa, barite, biocrystallization, biogeochemical cycles, celestite

## Abstract

Barium and strontium are often used as proxies of marine productivity in palaeoceanographic reconstructions of global climate. However, long-searched biological drivers for such correlations remain unknown. Here, we report that taxa within one of the most abundant groups of marine planktonic protists, diplonemids (Euglenozoa), are potent accumulators of intracellular barite (BaSO_4_), celestite (SrSO_4_), and strontiobarite (Ba,Sr)SO_4_. In culture, *Namystinia karyoxenos* accumulates Ba^2+^ and Sr^2+^ 42,000 and 10,000 times higher than the surrounding medium, forming barite and celestite representing 90% of the dry weight, the greatest concentration in biomass known to date. As heterotrophs, diplonemids are not restricted to the photic zone, and they are widespread in the oceans in astonishing abundance and diversity, as their distribution correlates with environmental particulate barite and celestite, prevailing in the mesopelagic zone. We found diplonemid predators, the filter-feeding zooplankton that produces fecal pellets containing the undigested celestite from diplonemids, facilitating its deposition on the seafloor. To the best of our knowledge, evidence for diplonemid biomineralization presents the strongest explanation for the occurrence of particulate barite and celestite in the marine environment. Both structures of the crystals and their variable chemical compositions found in diplonemids fit the properties of environmentally sampled particulate barite and celestite. Finally, we propose that diplonemids, which emerged during the Neoproterozoic era, qualify as impactful players in Ba^2+^/Sr^2+^ cycling in the ocean that has possibly contributed to sedimentary rock formation over long geological periods.

## INTRODUCTION

Although in most environments strontium (Sr) and barium (Ba) are present in trace amounts, they can be accumulated in substantial quantities by some organisms (1, 2). Depending on their environmental availability, these elements are mostly taken up nonselectively together with Ca^2+^ (3, 4). The soluble form of Ba^2+^ is typically toxic for animals (e.g., use of rodenticides) due to its capacity to block K^+^ channels, while insoluble BaSO_4_ acts as a common contrast agent in medical radio-imaging (5). In contrast, soluble Sr^2+^ is not harmful, with the exception of the radioactive isotope ^90^Sr^2+^ occurring as a nuclear contaminant that accumulates in marine biota and sediments (6). Indeed, in some algae, Sr^2+^ can almost fully replace Ca^2+^ without any discernible deleterious effects (7). In humans, Sr^2+^ treatment of osteoporosis is used to prevent fractures (8). Moreover, predictions concerning climate change stress the increased relevance of higher environmental mobilization of Sr^2+^ and Ba^2+^ due to enhanced solubility upon marine acidification (9). Apart from chemical precipitation treatments of radioactive ^90^Sr^2+^ and toxic Ba^2+^, there have been new attempts for bioremediation using cyanobacteria, algae, and fungi (1, 2, 6, 10).

In marine environments, microorganisms accumulate more Sr^2+^ than Ba^2+^, possibly due to higher solubility and availability, i.e., the concentration of Sr^2+^ is around 88 μM compared to 40 to 150 nM Ba^2+^ (11, 12). In protists, Sr^2+^ is mostly present in the form of celestite (also referred to as celestine; SrSO_4_) and strontianite (SrCO_3_), while Ba^2+^ forms barite (BaSO_4_) or witherite (BaCO_3_) (13, 14). Moreover, Ba^2+^ and Sr^2+^ commonly substitute for each other in various ratios to form strontiobarite and baritocelestite (Ba,Sr)SO_4_ (15). Celestite with traces of Ba^2+^ is well known for forming the complex skeletons of acanthareans (16). Intracellular barite crystals form statoliths of some freshwater charophyte algae and statocysts of marine ciliates, in which they likely play a role in graviperception (13, 17, 18). Haptophytes and foraminiferans form intracellular barite crystals with trace amounts of Sr^2+^ (19, 20), while strontianite and witherite occur in microalga *Tetraselmis* (21) and coccolithophorids (14, 22). The exact role of these crystalline inclusions remains unknown.

Marine Ba^2+^ and Sr^2+^ are frequently correlated with particulate organic carbon in the water column and sediments on the sea floor, indicating that microorganisms are capable of accumulating these elements (11, 23, 24), yet the celestite-rich skeletons of acanthareans dissolve during sedimentation (25). Ba^2+^ and Sr^2+^ carbonates and phosphates known from coccolithophorids and bacteria, respectively, contribute to the cycling of these elements with possible conversion to sulfates in the process of diagenesis (10, 14, 22, 26). In addition, barite, strontiobarite and celestite crystals are frequently found associated with fecal pellets, which contribute to the sedimentation of particulate Ba^2+^ and Sr^2+^ to the sea floor (24). However, until now, abundant planktonic organisms capable of selective intracellular accumulation of both Ba^2+^ and Sr^2+^ sulfates have not been identified (12, 13). The substantial work of Dehairs et al. (24) presents a series of evidence pointing to the biogenic origin of barite/celestite microcrystals, including micrographs of environmental microcrystals covered by desiccated cellular organic matter. Variable composition of marine suspended microcrystalline sulfates are commonly ascribed to barite with minor admixtures of Sr^2+^ alongside 10 to 30% of crystals dominated by celestite (24). Such variability is most plausibly explained by active biological catalysis (24). Despite the well-documented evidence-based predictions of the biogenic origins of barite and celestite minerals in the oceans (24, 27), the lack of organisms responsible for their production led to the gradual focus on microenvironment-mediated precipitation, stepping away from consideration of their biological origin (28).

Here, we show that diplonemids (Diplonemea, Euglenozoa), a group of biflagellated heterotrophic protists (29–31), are capable of massive intracellular accumulation of Sr^2+^ and Ba^2+^. Specifically, three cultivable diplonemids accumulate celestite and sometimes barite crystals in intracellular concentrations of Sr^2+^ much greater than in other organisms (10, 19). In the world’s oceans, diplonemids have only recently been recognized as omnipresent and one of the most diverse and abundant groups of microeukaryotes (comparable to microalgae), with a prevalence within the mesopelagic protist community (32–34). Although relatively rare, they are present in freshwater bodies as well (35). We analyzed their crystalline inclusions by a range of complementary approaches and discuss here their possible biological functions and role in biogeochemical cycles.

## RESULTS

### Light microscopy and analysis of crystals by Raman microscopy.

**FIG 1:**
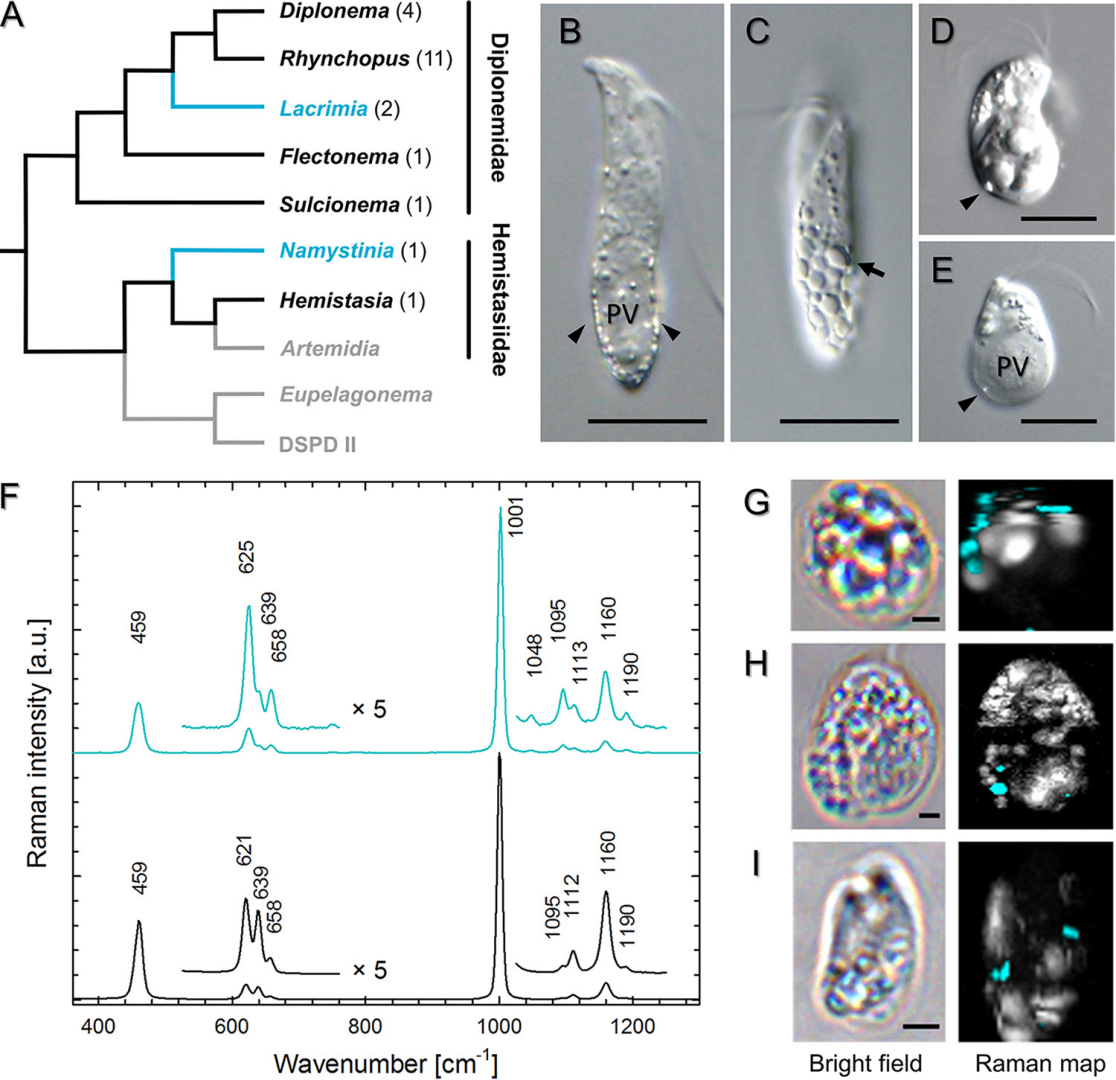
Distribution of celestite in diplonemids, based on Raman microscopy analysis. (A) Phylogenetic tree of diplonemids based on 29 with genera containing celestite (blue), other screened genera (black), and unexamined clades (gray). (B to E) DIC micrographs of *N. karyoxenos* (B and C), *Lacrimia* sp. YPF1808 (D), and *L. lanifica* (E) with celestite crystals marked by arrowheads; arrow points to a large polygonal crystal. Scale bar, 10 μm. (F) Raman spectra of biogenic celestite crystals found in diplonemid cells (blue) and celestite mineral (black). (G to I) Raman chemical maps of *N. karyoxenos* (G), *Lacrimia* sp. YPF1808 (H), and *L. lanifica* JW1601 (I) with celestite in blue and other cytoplasmic contents in white. Scale bar, 2 μm.

To determine the chemical composition of biogenic crystals directly within intact cells, Raman microscopy, a vibrational spectroscopic method sensitive to molecular composition, was used. Out of 21 strains belonging to 15 diplonemid species, three members of the distantly related genera *Lacrimia* and *Namystinia*, represented by *Lacrimia* sp. YPF1808, *Lacrima lanifica*, and *Namystinia karyoxenos*, were shown to possess celestite crystals (Fig. 1A). Their Raman spectra were congruent with the spectra of mineral celestite and chemically prepared precipitates of SrSO_4_ (Fig. 1F), matching also Raman spectra of celestite reported elsewhere (36). Due to the countercation sensitivity of the position of the most intense Raman band at around 1,000 cm^−1^ belonging to the symmetric ν_1_ vibrational mode of the SO_4_^2–^ tetrahedron, biogenic celestite could be unambiguously identified as SrSO_4_ and was easily distinguishable from barite, baritocelestite, gypsum (CaSO_4_), or calcite (CaCO_3_) (see Fig. S1 in the supplemental material). Small relative-intensity nuances of other Raman bands of biogenic celestite from various cells (Fig. S2) can be explained by differences in crystal structures (lattice defects), trace admixtures of Ba^2+^, and/or orientations of the crystals, as these are present also in the spectra of mineral reference and chemical precipitates.

10.1128/mbio.03279-22.1 FIG S1 Typical Raman spectrum of biogenic celestite crystals compared with the spectra of chemically prepared barite, the equimolar precipitate of SrSO_4_ and BaSO_4_, gypsum (CaSO_4_), and calcite (CaCO_3_). Each complex of SO_4_^2–^ with Sr^2+^, Ba^2+^, or Ca^2+^ can be unambiguously identified by the position of the most intense Raman band at around 1,000 cm^−1^, belonging to the symmetric ν_1_ vibrational mode of oxygen atoms within the SO_4_^2–^ tetrahedron. Download FIG S1, PDF file, 0.2 MB. Copyright © 2023 Pilátová et al. 2023 Pilátová et al. https://creativecommons.org/licenses/by/4.0/ This content is distributed under the terms of the Creative Commons Attribution 4.0 International license.

10.1128/mbio.03279-22.2 FIG S2 Typical Raman spectra (A to E) of biogenic celestite crystals found in various diplonemid cells. In terms of Raman frequencies, the spectra were virtually identical; however, minor spectral variability in the relative intensities of Raman bands was observed. Similar variability was also observed for mineral celestite and chemically prepared SrSO_4_ precipitates. Download FIG S2, PDF file, 0.1 MB. Copyright © 2023 Pilátová et al. 2023 Pilátová et al. https://creativecommons.org/licenses/by/4.0/ This content is distributed under the terms of the Creative Commons Attribution 4.0 International license.

When *N. karyoxenos* was examined by light microscopy with differential interference contrast (DIC), the crystalline structures appeared as small birefringent particles moving fast by Brownian motion (Fig. 1B and C; Movie S1). They were most prominent within the enlarged lacunae, which are peripheral membrane-bounded compartments positioned directly beneath the subpellicular microtubular corset (Movie S1), following the addition of 0.1% (wt/vol) formaldehyde. Within a single culture, the size and quantity of crystals inside the cells ranged from a few small particles (Fig. 1B) up to multiple large, tightly packed, polygonal crystals reflecting the shape of orthorhombic prisms (Fig. 1C). The crystalline particles of *Lacrimia* sp. YPF1808 and *L. lanifica* were far less prominent under the light microscope than those of *N. karyoxenos*. However, large crystals were visible around the posterior vacuole with DIC (Fig. 1D and E; Movie S1) and under polarized light (Movie S1).

10.1128/mbio.03279-22.8 MOVIE S1 *N. karyoxenos* and *Lacrimia* sp. YPF1808 under bright-field DIC and polarization microscopy showing fast-moving light-polarizing celestite crystals in lacunae marked by arrows. Download Movie S1, MOV file, 12.6 MB. Copyright © 2023 Pilátová et al. 2023 Pilátová et al. https://creativecommons.org/licenses/by/4.0/ This content is distributed under the terms of the Creative Commons Attribution 4.0 International license.

### Morphology, localization, and elemental analysis of intracellular crystals.

**FIG 2:**
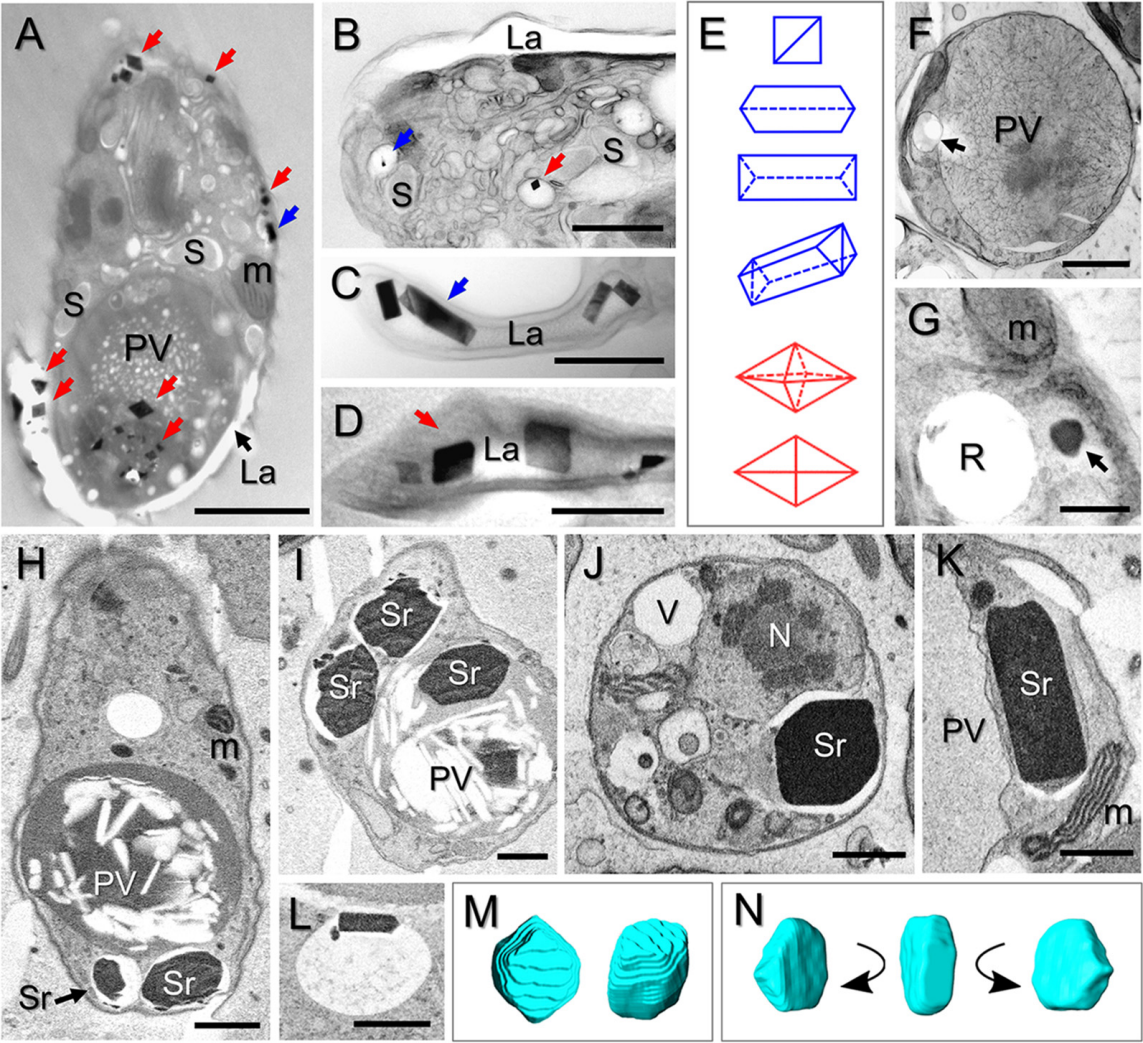
Crystal structures of naturally occurring celestite with possible barite admixtures in diplonemids. (A to E) TEM images of semithin sections of *N. karyoxenos* with longitudinally sectioned cell showing bipyramidal (red arrows) and prismatic (blue arrow) crystals inside peripheral lacunae and posterior vacuole (A); crystals contained within small vacuoles (B); prismatic (C) and bipyramidal (D) crystals inside lacunae; and a schematic representation of prismatic (blue) and bipyramidal (red) crystals (E). (F and G) TEM images of semithin sections of *L. lanifica* JW1601, with cells cross-sectioned through a posterior vacuole. An arrow points to a small membrane-bounded compartment with the hole left after a dropped-out crystal (F), and another arrow indicates a crystal inside a membrane-bounded compartment and the rupture introduced by a crystal during sectioning (G). (H to L) SBF-SEM images of *Lacrimia* sp. YPF1808 showing celestite crystals inside membrane-bounded compartments and the large posterior vacuole (I). (M and N) 3D reconstructions of celestite correspond to the images in panels J and K, respectively, in shape of rhombic prism (M) or asymmetric tabular prismatic crystal with pyramidal and pedial terminations (N). PV, posterior vacuole; M, mitochondrion; S, endosymbiotic bacteria; La, lacuna lumen; R, rupture; Sr, celestite crystal; N, nucleus. Scale bar, 2 μm (A and F), 1 μm (B and H to K), and 500 nm (C, D, and G).

Examination with light, Raman, transmission electron microscopy (TEM), and serial block-face scanning electron microscopy (SBF-SEM) showed that the crystalline inclusions in two clades of diplonemids differed in their localization and shapes. In semithin resin-embedded sections of *N. karyoxenos*, numerous orthorhombic prismatic and bipyramidal crystals were localized mostly inside the lacunae (Fig. 2A, C, and D), with a preference towards the cell posterior. Occasionally, crystals were found inside the large posterior vacuole (Fig. 2A) and in smaller vacuoles scattered throughout the cytoplasm (Fig. 2A and B). Only small crystals could be seen in semithin sections, while bigger crystals dropped out, leaving empty crystal-shaped holes. Due to frequent rupturing, it was not possible to visualize celestite crystals in semithin epoxy resin sections of *Lacrimia* species. Thus, we used the SBF-SEM approach, which showed that the celestite crystals of *Lacrimia* sp. YPF1808 appeared mostly in small membrane-bounded compartments with electron-transparent matrix (Fig. 2H to L) adjacent to the large posterior vacuole (Fig. 2H, I, and K). Three-dimensional (3D) reconstruction revealed that each of these compartments contained one crystal of variable size (Movie S2). Less frequently, crystals were found inside the posterior vacuole (Fig. 2I) or in compartments localized near the anterior flagellar pocket (Movie S2), while they were absent from the cytoplasm and other organelles. The crystals had a shape of rhombic prisms (Fig. 2J and M) or asymmetric tabular prismatic structures with pyramidal and pedial terminations (Fig. 2K and N). Although in *L. lanifica* the celestite crystals were mostly lost from the TEM sections, the positions of holes and ruptures within them and the analysis by Raman microscopy showed similar localizations and sizes of the crystals as those of *Lacrimia* sp. YPF1808 (Fig. 2D, E, H, and I). Likewise, the membrane-bounded compartments were positioned around the posterior vacuole (Fig. 2F), with small asymmetric flattened crystals preserved only occasionally in TEM sections (Fig. 2G).

10.1128/mbio.03279-22.9 MOVIE S2 Visualization of serial sections through a celestite-containing cell of *Lacrimia* sp. YPF1808 taken by SBF-SEM with celestite crystals marked by arrows. PV, posterior vacuole; N, nucleus; FP, flagellar pocket. 3D reconstruction of a single cell via SBF-SEM shows the cytoplasm in yellow, a large posterior vacuole in orange, and celestine crystals in cyan. Download Movie S2, MOV file, 12.9 MB. Copyright © 2023 Pilátová et al. 2023 Pilátová et al. https://creativecommons.org/licenses/by/4.0/ This content is distributed under the terms of the Creative Commons Attribution 4.0 International license.

**FIG 3:**
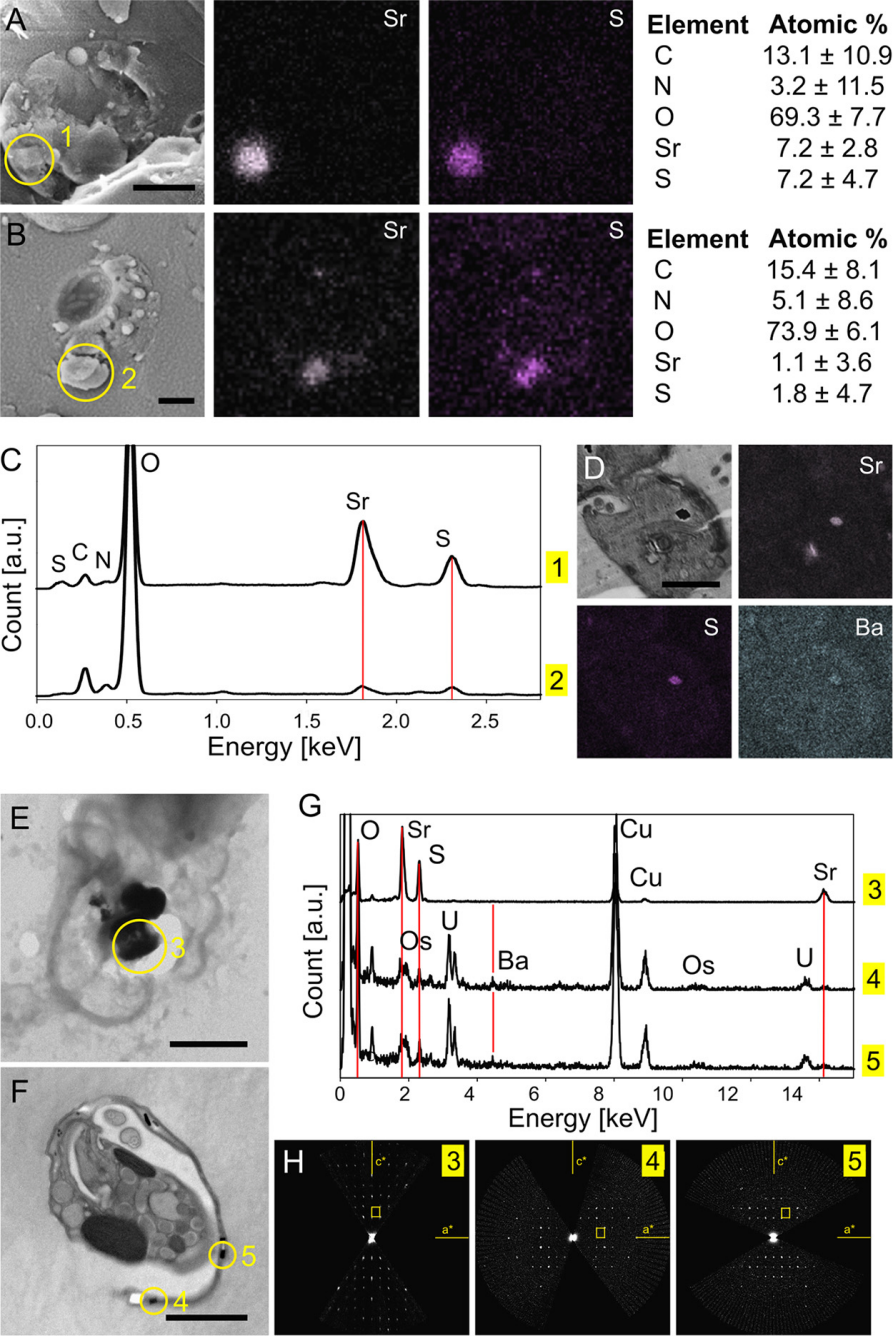
Elemental analysis of diplonemids. (A to C) Cryo-SEM-EDX images of *Lacrimia* sp. YPF1808 (A) and *N. karyoxenos* (B), complemented with EDX elemental spectral analysis (C) obtained from the area marked by circles 1 and 2, respectively. (D) SEM-EDX images of *Lacrimia* sp. YPF1808 showing the presence of S, Sr, and Ba. (E to G) TEM analysis of celestite microcrystals in a micrograph of a dried cell of *L. lanifica* (E), of a semithin section of *N. karyoxenos* (F), or EDX spectra (G) from the corresponding areas marked 3 to 5, with red lines highlighting the positions of O, Sr, Ba, and S. Cu and C originated from the support grid, and Os and U originated from staining compounds. (H) Electron diffraction for h0l-oriented sections through the 3D ED data sets from the corresponding areas shown in panels E and F. The celestite unit cell is displayed as a yellow rectangle. Scale bar, 2 μm.

The presence of celestite crystals (Fig. 1 and 2) was further confirmed by elemental analysis using energy-dispersive X-ray (EDX) spectroscopy in the cryo-SEM-EDX mode of freeze-fractured *Lacrimia* sp. YPF1808 (Fig. 3A and C; Fig. S3) and *N. karyoxenos* cells (Fig. 3B and C; Fig. S4) and by TEM-EDX of whole air-dried cells of *L. lanifica* (Fig. 3E). Atomic percentages estimated by cryo-SEM-EDX analysis were 7.2% Sr and 7.2% sulfur (S), compared to 1.1% Sr and 1.8% S in *Lacrimia* sp. YPF1808 and *N. karyoxenos*, respectively. The dominance of C, N, and O atoms can be explained by the presence of ice and signals from other cellular contents obtained from deeper and/or surrounding areas.

10.1128/mbio.03279-22.3 FIG S3 Cryo-SEM-EDX analysis of *Lacrimia* sp. YPF1808 showing the elemental analysis of freeze-fractured samples. (A) SEM micrograph; (B) merged image of the most common elements; (C) merged image of all elements present; (D) map of carbon K line; (E) map of nitrogen K line; (F) map of oxygen K line; (G) map of magnesium K line; (H) map of silicon K line; (I) map of strontium L line; (J) map of phosphorus K line; (K) map of sulfur K line; (L) map of chlorine K lines; (M) map of calcium K line; (N) map of barium L line. (O) Pie chart of the most common elements detected in the area with particular pixel counts as depicted in panel B: oxygen and carbon K lines (blue), oxygen K line (red), oxygen K line, strontium L line, sulfur K line (yellow). Scale bar, 10 μm. Download FIG S3, PDF file, 0.1 MB. Copyright © 2023 Pilátová et al. 2023 Pilátová et al. https://creativecommons.org/licenses/by/4.0/ This content is distributed under the terms of the Creative Commons Attribution 4.0 International license.

10.1128/mbio.03279-22.4 FIG S4 Cryo-SEM-EDX analysis of *N. karyoxenos* freeze-fractured samples. (A) SEM micrograph; (B) merged image of the most common elements; (C) merged image of all elements present; (D) map of carbon K line; (E) map of nitrogen K line; (F) map of magnesium K line; (G) map of silicon K line; (H) map of strontium L line; (I) map of phosphorus K line; (J) map of sulfur K line; (K) map of chlorine K lines; (L) map of calcium K line. (M) Pie chart of the most common elements detected in the area with particular pixel counts as depicted in panel B: oxygen and carbon K lines (blue), oxygen K line (dark red), oxygen and carbon K lines (yellow). Scale bar, 10 μm. Download FIG S4, PDF file, 0.1 MB. Copyright © 2023 Pilátová et al. 2023 Pilátová et al. https://creativecommons.org/licenses/by/4.0/ This content is distributed under the terms of the Creative Commons Attribution 4.0 International license.

The identities of celestite crystals in *Lacrimia* sp. YPF1808 (1 μm-thick sections from resin blocks used for SBF-SEM) and *N. karyoxenos* (250 nm-thick resin sections examined by TEM) were confirmed by SEM-EDX and TEM-EDX, respectively (Fig. 3D, F, and G; Fig. S5). Additionally, a significant amount of Ba^2+^ was detected in the crystals from *N. karyoxenos*. Crystallographic analysis by electron diffraction showed that the diffraction of measured crystals corresponded to celestite structure (isostructural with BaSO_4_) with space group Pnma and lattice parameters *a *= 8.3 Å, *b *= 5.3 Å, and *c *= 6.8 Å in *L. lanifica*. Larger lattice parameters (*a *= 8.7 Å, *b *= 5.5 Å, *c *= 7.1 Å) were observed in *N. karyoxenos*, which may be explained by the replacement of Sr^2+^ with larger Ba^2+^ in the structure of celestite.

10.1128/mbio.03279-22.5 FIG S5 SEM-EDX analysis of *Lacrimia* sp. YPF1808 showing the elemental composition of samples used for 3D reconstruction by SBF-SEM. (A) Electron micrograph; (B) merged image of the most common elements; (C) merged image of all elements present; (D) map of carbon K line; (E) map of oxygen K line; (F) map of strontium L line; (G) map of calcium L line; (H) map of barium M line; (I) map of osmium M line; (J) map of nitrogen K line; (K) map of sodium K line; (L) map of sulfur K lines. (M) Pie chart of the most common elements detected in the area with particular pixel counts as depicted in panel B: *, oxygen K line/calcium L line; **, oxygen K line/calcium L line/nitrogen K line; ***, oxygen K line/calcium L line/nitrogen K line/strontium L line/osmium M line. Scale bar, 2 μm. Download FIG S5, PDF file, 0.1 MB. Copyright © 2023 Pilátová et al. 2023 Pilátová et al. https://creativecommons.org/licenses/by/4.0/ This content is distributed under the terms of the Creative Commons Attribution 4.0 International license.

### Quantitative analysis by ICP-MS and SBF-SEM.

**FIG 4:**
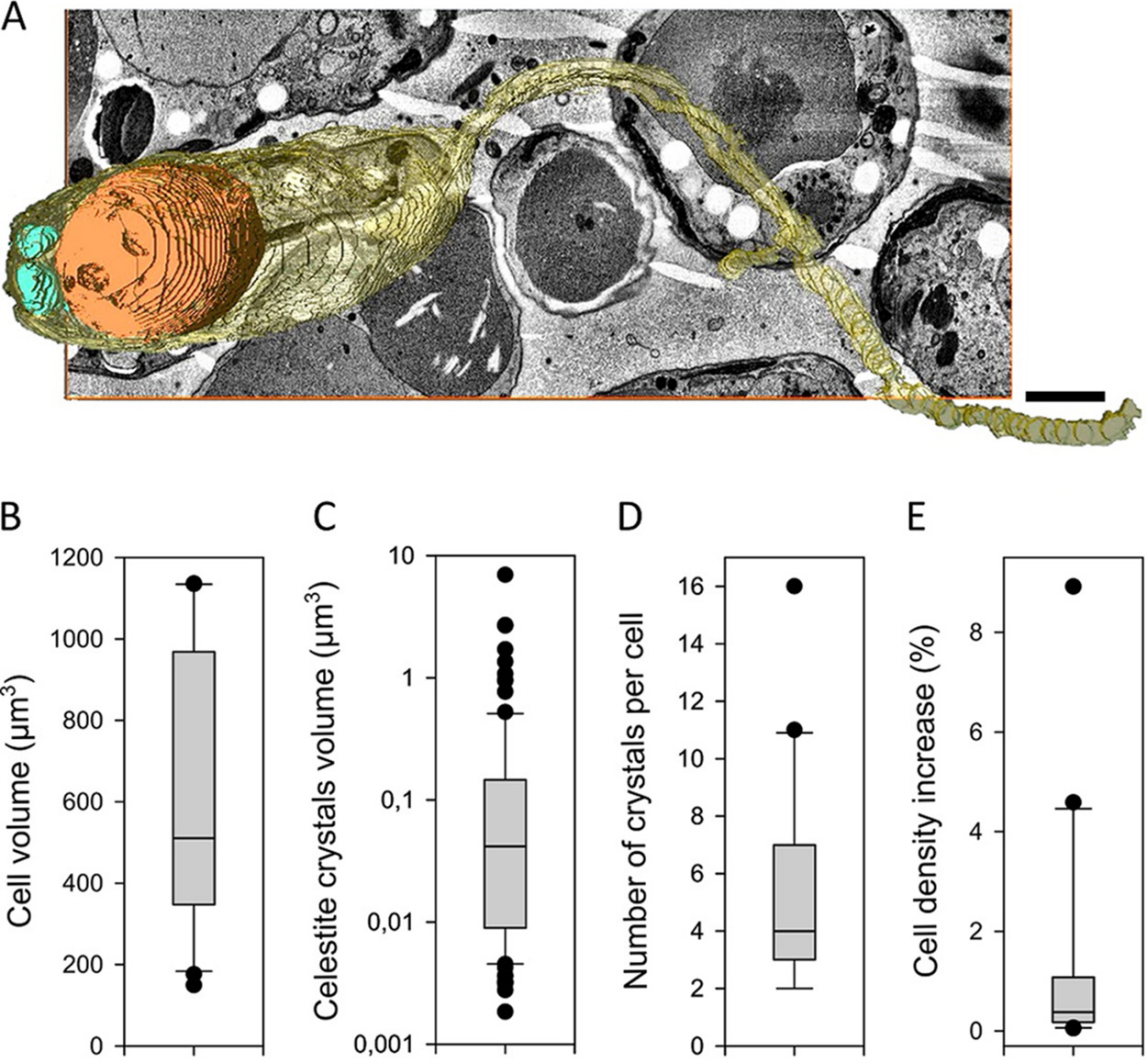
SBF-SEM images of celestite crystals in *Lacrimia* sp. YPF1808. (A) 3D reconstruction of a cell. Cytoplasm is shown in yellow, large posterior vacuole is in orange, and celestine crystals are in cyan. Scale bar, 1 μm. Descriptive analysis of measured data is for cells (*n* = 20) and crystals (*n* = 106). (B) Distribution of cell volumes. (C) Distribution of celestite crystal volumes on a log scale. (D) Number of celestite crystals per cell. (E) Impacts of celestite crystals on cell density; boxplots show medians and quartiles, and whiskers show the range from minimum to maximum values excluding outliers, represented by single data points.

SBF-SEM-based 3D reconstructions of *Lacrimia* sp. YPF1808 (Movie S2) showed the presence of celestite crystals in all 20 analyzed cells, ranging from 2 to 16 celestite particles per cell (Fig. 4D; Table S2). In total, more than 100 crystals were analyzed, with a volume ranging from 0.017 to 7 μm^3^ (Fig. 4C; Table S2). The impacts of the measured celestite contents on the overall cell density ranged from 0.05% to 9%, with an average of 1.3 ± 0.5% (Fig. 4E; Table S2). The calculations were based on the measured volumes, known density of celestite (3.9 g·cm^−3^), and common cellular densities of 0.985 to 1.156 g·cm^−3^ reported elsewhere (37).

10.1128/mbio.03279-22.7 TABLE S2 ICP-MS data and calculations with Sr and Ba amounts in 6 diplonemid species (Table S2A), calculations of concentration of Ba and Sr inside the cells relative to the concentration in the surrounding medium (Table S2B), and volumetric measurements of diplonemid *Lacrimia* sp. YPF1808 (Table S2C) – based on SBF-SEM 3D reconstruction with the calculation of theoretical cell density based on literature, calculations of cell and crystal densities and theoretical sedimentation rates assumed based on Stokes' law. Download Table S2, PDF file, 1.8 MB. Copyright © 2023 Pilátová et al. 2023 Pilátová et al. https://creativecommons.org/licenses/by/4.0/ This content is distributed under the terms of the Creative Commons Attribution 4.0 International license.

The lack of celestite crystals in other analyzed species (*Diplonema japonicum*, *Paradiplonema papillatum*, and *Rhynchopus* sp. YZ270) was consistent with the minute ^88^Sr content measured by ICP-MS. The high values of ^88^Sr in *N. karyoxenos*, *Lacrimia* sp. YPF1808, and *L. lanifica* corresponded to the abundance of intracellular crystals detected by Raman microscopy, TEM, and SBF-SEM. Since direct measurement of the dry mass was impossible due to the inevitable presence of salts from the medium, the elemental composition analysis by ICP-MS was calculated in atoms per cell or femtomoles per cell. To calculate Sr and Ba content per dry mass, the latter was subsequently estimated by quantitative phase imaging using holographic microscopy (Table 1). The ^88^Sr amounts ranged from 0.01 fmol·cell^−1^ in *P. papillatum* to 5,500 ± 570 fmol·cell^−1^ (mean ± standard deviation) in *N. karyoxenos*, corresponding to 340 ± 38 mg·g^−1^. *Lacrimia* sp. YPF1808 and *L. lanifica* were also potent ^88^Sr accumulators, with 370 ± 58 fmol·cell^−1^ (130 ± 25 mg·g^−1^) and 54 ± 8 fmol·cell^−1^ (64 ± 13 mg·g^−1^), respectively. Depending on the species, the intracellular concentration of ^88^Sr was 1,200 to almost 10,000 times higher than in the surrounding medium (Tables 1 and S2).

**Table 1:** ICP-MS quantification of ^88^Sr and Ba in diplonemids^*a*^

Species	*D. japonicum*	*P. papillatum*	*Rhynchopus* sp. YZ270	*L. lanifica* JW1601	*Lacrimia* sp. YPF1808	*N. karyoxenos*
Number of cells per ml in culture	2.1·10^5^ ± 9.2·10^3^	2.2·10^6^ ± 2.1·10^5^	8.1·10^5^ ± 2.6 ·10^4^	7.6·10^5^ ± 2.5·10^4^	3.7·10^6^ ± 1.7·10^5^	1.33·10^6^ ± 5.9·10^4^
Dry weight of cell (pg)	109 ± 2.9	68.0 ± 2.0	74.2 ± 2.7	74.1 ± 3.5	248.1 ± 8.2	1,421 ± 11
Sr (fmol·cell^–1^)	0.22 ± 0.01	0.013 ± 0.001	0.08 ± 0.02	54.4 ± 8.4	366 ± 58	5,530 ± 570
Sr (mg·g^–1^)	0.17 ± 0.10	0.02 ± 0.01	0.10 ± 0.07	64 ± 13	129 ± 25	340 ± 38
*p*	a	b	a	c	d	e
SrSO_4_ (mg·g^–1^)	n/a	n/a	n/a	135	271	715
Concentration of Sr (folds)*^b^	n/a	n/a	n/a	1,520 ± 680	1,240 ± 550	10,000 ± 3,300
Ba (fmol·cell^–1^)	n/a	n/a	n/a	2.0 ± 0.5	65 ± 10	1,200 ± 130
Ba (mg·g^–1^)	n/a	n/a	n/a	3.7 ± 1.1	35.8 ± 6.8	116 ± 14
*p*	n/a	n/a	n/a	a	b	c
BaSO_4_ (mg·g^–1^)	n/a	n/a	n/a	6.3	61	198
Concentration of Ba (folds)*^b^	n/a	n/a	n/a	1,130 ± 660	4,300 ± 1,900	42,000 ± 14,000
Total Ba,Sr(SO_4_) (mg·g^–1^)	n/a	n/a	n/a	141	332	913

a The dry weight was measured by quantitative phase imaging (n = 150 cells). Results of ICP-MS are displayed as mean values of biological triplicates with standard error of the mean. All figures are rounded to two significant numbers. The amounts of ^88^Sr and Ba per dry mass (mg·g^−1^) was logarithmically transformed and statistically analyzed by one-way ANOVA *p* < 0.001 (F = 1747.8 and F = 88.6; total degree of freedom: df = 17 and df = 8, respectively), with Tukey’s post-hoc test significant differences on the level *p* < 0.05 in column *p*.

b *, calculated as atoms**·**cell^−1^ based on analyzed cell pellets relative to the theoretical value in atoms**·**cell^−1^ originating from the culture medium alone (8 mg·l^−1^ Sr^2+^ and 0.64 mg·l^−1^ Ba^2+^ – as listed in SI Appendix Table S1); in *N. karyoxenos*, the amount of accumulated Ba exceeds total Ba available per volume of culture medium due to repeated passages of pelleted cells into fresh medium. n/a, not analyzed.

10.1128/mbio.03279-22.6 TABLE S1 ICP-MS analysis of seawater growth medium and artificial medium without the addition of sulfates, Ba^2+^, and Sr^2+^ sources (the latter was used for culturing cells in Ba^2+^ loading experiments and as a rinsing solution for the preparation of ICP-MS samples; means of three technical replicates ± standard deviations are shown). Download Table S1, PDF file, 0.04 MB. Copyright © 2023 Pilátová et al. 2023 Pilátová et al. https://creativecommons.org/licenses/by/4.0/ This content is distributed under the terms of the Creative Commons Attribution 4.0 International license.

Compared to the massive accumulation of ^88^Sr in diplonemids, the naturally co-occurring Ba was present in much lower amounts (Table 1 and S2), slightly above the detection limit of TEM-EDX analysis (Fig. 3G), possibly reflecting a 12.5 times lower Ba^2+^ concentration in the seawater growth medium (Table S1). Nevertheless, the cells concentrated Ba^2+^ on average 1,000 to over 42,000 times above the level in the growth medium (Table S1), reaching 1,200 ± 130 fmol·cell^−1^ (120 ± 14 mg·g^−1^) in *N. karyoxenos*, with lower values in *Lacrimia* sp. YPF1808 (65 ± 10 fmol·cell^−1^, or 36 ± 7 mg·g^−1^) and *L. lanifica* (2 ± 1 fmol·cell^−1^, or 4 ± 1 mg·g^−1^). Altogether, (Ba,Sr)SO_4_ accumulation reached 91%, 33%, and 14% of dry weight in *N. karyoxenos*, *Lacrimia* sp. YPF1808, and *L. lanifica*, respectively. All strains showed significantly different levels of accumulated Sr or Ba contents (Table 1). Although the concentration of Ba^2+^ in our experiment was 30 to 45 times higher than in nature, the stoichiometry per cell was 5 orders of magnitude lower than in the oceans (Table 1).

### Ba^2+^ loading experiments and elimination of Sr^2+^ and Ba^2+^ from the medium.

**FIG 5:**
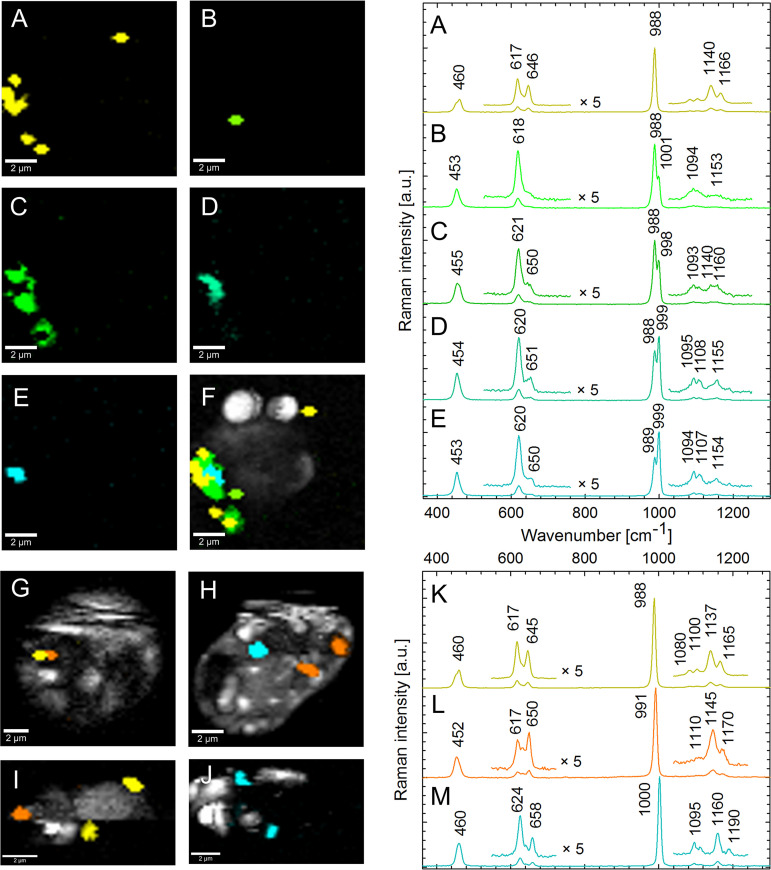
Raman maps and spectra of barite and celestite crystals in diplonemids cultivated with equimolar amounts of Sr^2+^ and Ba^2+^ in the medium. (A to F) A single cell of *N. karyoxenos* containing cocrystallized fractions dominated by celestite in the central part (E; cyan) with gradually overlapping barite (B to D; shades of green) toward the periphery of its pure fraction (A; yellow), and the merged Raman map of of panels A to E (F). The limited spatial resolution of Raman microscopy did not allow distinguishing conglomerates of pure-species microcrystals from a single crystal with a variable elemental composition. (G to M) Two cells of *Lacrimia* sp. YPF1808 (G and H) and two cells *L. lanifica* (I and J) contained pure barite (K; yellow) and celestite (M; blue) and homogenously mixed crystals of (Ba,Sr)SO_4_ (L; orange).

Under our cultivation conditions, Ba^2+^ was 20 times less abundant than Sr^2+^ (Table S1), which resulted in formation of celestite with Ba^2+^ admixtures in the examined species. In oceans, the proportion is even 30 times lower (11, 12). Here, we tested whether increases in Ba^2+^ content would impact the biomineralization process by cultivation in artificial seawater loaded with equimolar amounts of Ba^2+^ and Sr^2+^ to 88 μM. To prevent spontaneous precipitation of barite, we supplemented sulfates with NaCl to maintain the osmolarity of the medium. This resulted in the formation of all mineral combinations: pure barite (Raman marker at 988 cm^−1^), celestite (1,000 cm^−1^), and mixed forms of (Ba,Sr)SO_4_ (991 cm^−1^; strontiobarite or baritocelestite) (Fig. 5), revealing that diplonemids do not show a strong preference toward the accumulation of either element.

Both *L. lafinica* and *Lacrimia* sp. YPF1808 contained mostly mixed crystals of (Ba,Sr)SO_4_. In *N. karyoxenos*, pure celestite prevailed in the central part of crystals (999 cm^−1^) while barite dominated at the periphery (988 cm^−1^), and they gradually overlapped each other (Fig. 5C to F). Additionally, after several passages in the artificial medium without Ba^2+^ and Sr^2+^ (Table S1), barite and celestite were no longer detectable by Raman microscopy. The lack of Ba^2+^ and Sr^2+^ did not result in altered morphology or growth impairment. We did not observe any Ca^2+^- or Mg^2+^-containing crystals despite high concentrations of both elements in the medium.

### Feeding experiments.

**FIG 6:**
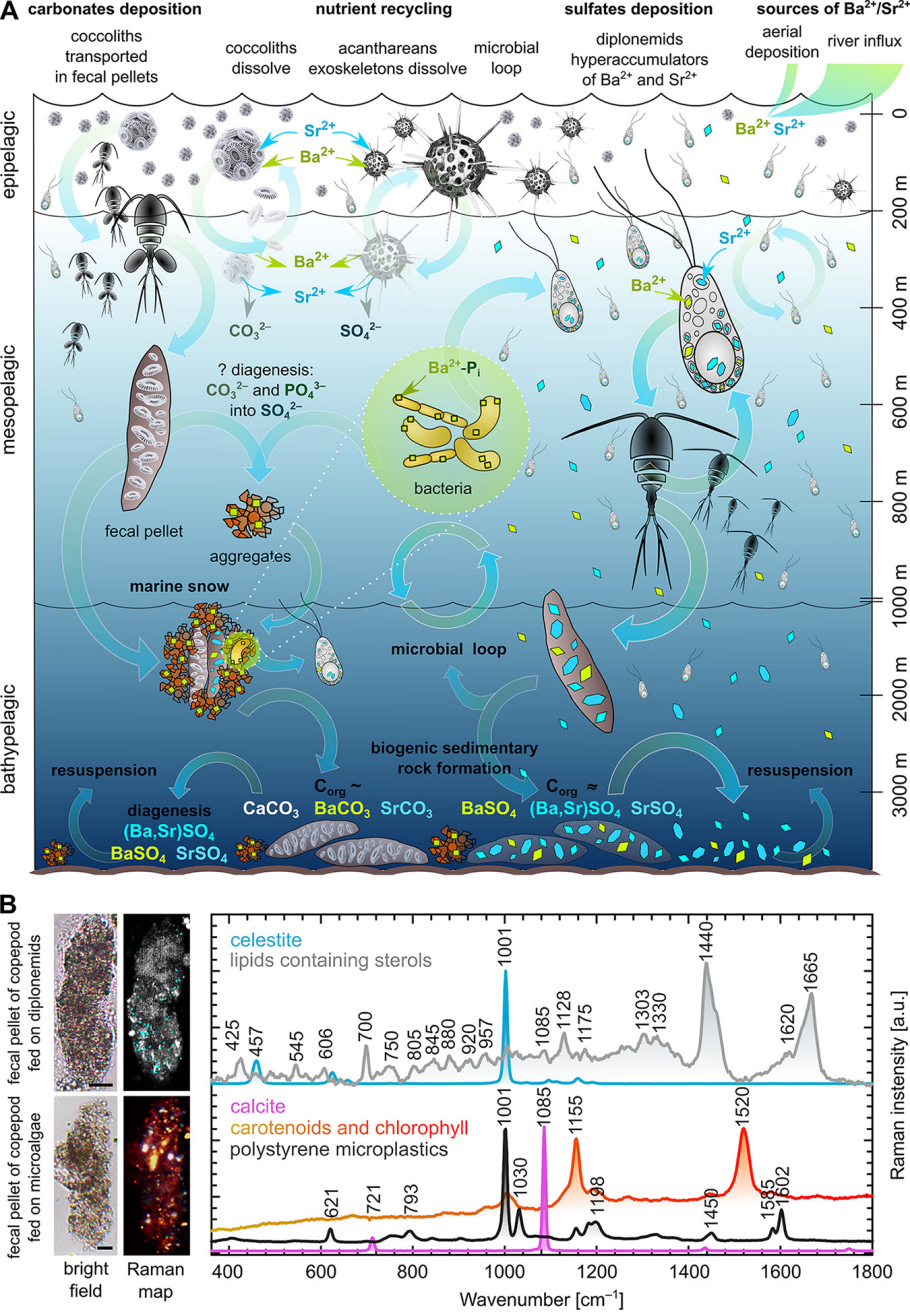
Schematic representation of the biological impact on Ba^2+^ and Sr^2+^ cycling in the oceans. (A) Hypothetical scenario of trace elements inlet, plankton uptake, recycling, and sedimentary deposition. The major source of trace elements is driven by river influx and less prominently by aerial deposition (9) and is mostly balanced by the same amount of total deposition in sediments, correlated with the total marine productivity and particulate organic matter deposition (24). A great proportion of these trace elements is being recycled by living organisms after release from dead cells. Acantharea (image adapted from referene 69) take up a substantial portion of Sr^2+^ and Ba^2+^, which are further recycled in upper 400 m (25). Coccolithophorids build their scales from calcium carbonate with minor amounts of Ba^2+^ and Sr^2+^ that are proportional to the seawater contents (22), being partially recycled upon dissolution or transported to the marine sediments in fecal pellets (24). The Ba^2+^ accumulated by bacteria sediments in the aggregates of marine snow (26) or is recycled. We highlighted diplonemids as potential players in the marine cycle of both elements and drivers of biogenic formation of celestite and barite crystals found in suspended matter everywhere in the world oceans (24), and they can also feed on bacteria or particulate organic matter or scavenge the dead bodies of zooplankton as major heterotrophic protists in the mesopelagic zone. (B) Raman microscopy analysis of fecal pellets produced by copepods experimentally fed with diplonemids, which contained celestite crystals (cyan) and undigested lipids, including sterols (gray). In the control samples of copepods fed with microalgae, fecal pellets contained undigested carotenoids, chlorophyll (yellow-orange-red), and calcite particles (pink), with unexpected particles of polystyrene microplastics (measured as a single spectrum [not shown on the Raman map]). Scale bars, 20 μm.

Fecal pellets are an important agent mediating sedimentation of biogenically accumulated minerals to the sea floor: large aggregates of crystals held together by undigested fecal organic matter enable their fast sinking, thus preventing dissolution of micrometer-sized crystals in the water column, which is undersaturated for barite and celestite (15, 24). To experimentally address whether zooplankton feeds on diplonemids and whether their celestite crystals are carried into fecal pellets, we incubated *N. karyoxenos* and *Lacrimia* sp. YPF1808 with freshly captured filter-feeding marine copepods *Centropages typicus*, *Temora longicornis*, and *Acartia* sp., starved for 12 h prior to the experiment. After 5-day cocultivation, we determined by Raman microscopy that the fecal pellets contained copious amounts of celestite derived from diplonemids (Fig. 6B). In the control system of the same population of copepods fed with freshly collected marine plankton, the fecal pellets contained undigested chlorophyll, carotenoids with remnants of lipids, calcite particles, and contaminating polystyrene microplastic particles, but lacked celestite crystals (Fig. 6B).

## DISCUSSION

The most studied biominerals in protists are extracellular calcite scales of haptophytes and silicate frustules of diatoms, while studies on intracellular mineral crystals are far less common (38). After more than a century since the skeletons of marine acanthareans and freshwater streptophytes were found to contain celestite (16) and barite (39), respectively, we have identified potent accumulators of Ba^2+^ and Sr^2+^ in an unexpected group of eukaryotes, the diplonemids.

The heterotrophic diplonemids are widespread in the oceans and, as recently described, in astonishing abundance and diversity (31, 33). Despite their abundance and extreme diversity, diplonemid flagellates remain a poorly known group of protists (34) that are abundant from the surface to the deep sea, with a wide peak in the mesopelagic zone (32, 33, 40, 41). The high capacity of intracellular Sr^2+^ and Ba^2+^ accumulation in some diplonemids outperforms that of any other reported organisms (10, 13, 21, 42–44). Indeed, while the intracellular concentration of Sr^2+^ in the most efficient accumulators known thus far (yeasts, desmids, and cyanobacteria) reaches a maximum of 220 mg·g^−1^ per dry weight (1, 10, 43), *N. karyoxenos* contains as much as 340 mg·g^−1^ Sr^2+^ together with 120 mg·g^−1^ Ba^2+^, which in the form of sulfate represents 90% of the cellular dry mass, pointing to the unique Sr^2+^ and Ba^2+^ accumulation capacity of this diplonemid, while both *Lacrimia* species are slightly less potent in this respect (Table 1).

Interestingly, when both trace elements are provided in equimolar concentrations, diplonemids form pure celestite and barite and/or mixed forms of (Ba,Sr)SO_4_, apparently not discriminating one element over the other. Hence, we explain the higher content of Sr^2+^ over Ba^2+^ inside the crystals by the higher availability of the former element in seawater. Although the mechanisms behind intracellular accumulation of Sr^2+^ and Ba^2+^ are largely unknown, it has been suggested that mineral crystals typically occur in membrane-bounded compartments or vacuoles, in which they are formed from supersaturated solutions via precisely regulated nucleation (13). The Sr^2+^ uptake and transportation within eukaryotic cells have been shown to occur via commonly present transporters of divalent cations, i.e., the Ca^2+^ uniporter and H^+^/Ca^2+^ antiporter (45, 46). The diplonemid nuclear genome is not yet available, but these transporters have been documented in the related kinetoplastid Trypanosoma brucei (47). Although the reported affinity to Ca^2+^ and Sr^2+^ is usually comparable (45, 46), some organisms including diplonemids clearly favor Ba^2+^ and Sr^2+^ over Ca^2+^ (10). When such vacuoles contain sulfate solutions, they may function as a “sulfate trap” for those cations that precipitate easily in the presence of sulfates (2). At the same time, we did not observe CaSO_4_ or any of its forms (gypsum, bassanite, anhydride, etc.), even though the concentration of Ca^2+^ in the cultivation medium or in the environment is several orders of magnitude higher than that of Sr^2+^ and Ba^2+^.

Densities of celestite and barite of 3.9 g·cm^−3^ and 4.5 g·cm^−3^, respectively, have been repeatedly reported as statoliths in ciliates or charophytic algae (13, 17, 18). In comparison to the seawater density of 1.03 g·cm^−3^ and typical cell density range between 0.985 and 1.156 g·cm^−3^, the heavy crystals may help maintain appropriate buoyancy by counterbalancing light lipid droplets (0.86 g·cm^−3^) (37, 48). Indeed, the impact of celestite crystals is substantial, since they may increase the overall density of *Lacrimia* sp. YPF1808 and *N. karyoxenos* by up to 9% and 27%, respectively (Table S2). According to Stokes’ law for small particles of low Reynolds numbers, the barite/celestite ballasting can significantly increase the sedimentary velocity for up to 50 to 200 m per month or 0.5 to 2 km per year (Table S2). Hence, while the function of biomineralization in diplonemids remains unknown, we speculate that they may benefit from gravitropic sensing, which would allow directed movement and/or enable passive sedimentation. Another intriguing impact of barite and celestite is associated with their propensity to strong absorption of UV and blue light (49). Hence, in surface waters, these minerals may contribute to UV protection. It is reasonable to assume that by forming celestite, protists adjust their inner osmolarity, the principle analogical to the formation of other cell inclusions, such as oxalate, calcite, or polyphosphate, that are either dissolved and osmotically active or crystallized or polymerized and osmotically inactive inside a vacuole (13, 50).

Celestite-forming acanthareans are considered key players in the upper 400 m of the ocean, yet do not contribute to the sedimentary rock formation, as their skeletons dissolve upon decay of their cells (25). Coccolithophorids and bacteria produce carbonates (44) and/or phosphates (26) of Ba^2+^/Sr^2+^, which can also be converted to sulfates either on the bacterial extracellular polymeric substances or in the microenvironment of decaying matter of marine snow aggregates in the process of diagenesis (26). In the chemical continuum between pure barite and celestite, the latter represents 10 to 30% (24), gradually decreasing, depending on the depth (11, 12). The majority of biogenic particulate barite and celestite is recycled by simple dissolution (25), microbial loop (26), or resuspension of sediments (24). However, the overall influx into the system is balanced by sedimentary deposition (9, 24), which might have a biological driver. Seminal work of Dehairs et al. (24) scrutinized all potential sources of particulate barite and celestite, and they did not find experimental support for either Ba^2+^ incorporation in siliceous plankton or precipitations on decaying organic matter in sulfate-enriched microenvironments. Hence, they ultimately favored the biogenic origin of particulate barite/celestite being hypothetically formed by microorganisms inhabiting the high-productivity mesopelagic zone (24) only to remain unknown since then. These predictions nicely correlate with our measurements in diplonemids, indicating that micron-sized celestite and sometimes barite crystals of variable Ba-Sr ratios (Fig. 2 to 5) are scattered throughout the water column of the world’s oceans, with the highest prevalence in the mesopelagic zone (32). Moreover, particulate barite/celestite is often found in fecal pellets and aggregates of marine snow, and finally, in the sediments (24, 27, 32). By providing celestite-containing diplonemids to filter-feeding copepods, we found undigested celestite in their fecal pellets (Fig. 6), the main transport system of micrometric biominerals into the sediments, although the majority is recycled (24). Thus, diplonemids may be involved in Ba^2+^/Sr^2+^ cycling and/or in sedimentary deposition of celestite or barite. Since these protists likely emerged during the Neoproterozoic era (590 to 900 million years ago [MYA]), overlapping with the Ediacaran period (51), their impact on biogenic marine sediments may cover several geological eras. The coccolithophores appeared around the same time as diplonemids, yet the onset of carbonate biomineralization has been timed to ~200 MYA (52).

As another ecological addition to the big picture of Ba^2+^/Sr^2+^ cycling, diplonemids have been shown to ingest bacteria as one of their sources of nutrition (30); if bacteria were loaded with Ba^2+^/Sr^2+^ in the form of (poly)phosphates, as reported elsewhere (26), diplonemids may further transform it into barite upon digestion. Additionally, diplonemids are likely to feed on the organic matter of marine snow providing preconcentrated Ba^2+^, in which case they may accumulate more Ba^2+^ than Sr^2+^. In principle, we experimentally supported such a scenario upon doping the cells with equimolar Ba^2+^ and Sr^2+^ concentrations (Fig. 5). Finally, we do not exclude that some species of diplonemids to be described in future would prefer Ba^2+^ over Sr^2+^ or that there are other as-yet-unknown microbial bioaccumulators of these trace elements.

Based on the ability of some diplonemids to store massive amounts of celestite and to lesser extent barite, we speculate that more as-yet-unknown diplonemid species may qualify as impactful players of Ba^2+^/Sr^2+^ flow through the food web, eventually influencing the sedimentary records.

## MATERIALS AND METHODS

### Cell cultures, cultivation, and light microscopy.

For all experiments, axenic cultures were grown in seawater-based Hemi medium (see Table S1 in the supplemental material) supplemented with 1% horse serum and 0.025 g/liter LB broth powder (53). An artificial seawater medium lacking Sr^2+^, Ba^2+^, and sulfates was prepared from 288 mM NaCl, 8 mM KCl, 718 mM KBr, 100 mM MgCl_2_, 12 mM CaCl_2_, 40 mM HBO_3_, and 60 mM NaF, supplemented with 1% (vol/vol) heat-inactivated horse serum (Sigma-Aldrich) and 25 mg LB broth powder (Amresco). The medium was used as rinsing solution for preparation of ICP-MS samples and cell microcrystal depletion to be measured via quantitative phase imaging (QPI) (see below). For Ba^2+^ loading experiments, BaCl_2_ was added in equimolar amounts with respect to naturally occurring (88 μM) Sr^2+^ (12).

Axenic clonal cultures of 17 species of diplonemids were grown either at 27°C (*Paradiplonema papillatum* ATCC 50162), 22°C (*Namystinia karyoxenos* YPF1621), or 13°C (of *D. aggregatum* YPF1605, *D. japonicum* YPF1604, *Flectonema* sp. DT1601, *Hemistasia phaeocysticola*, *Lacrimia lanifica* JW1601, *Lacrimia* sp. YPF1808, *Rhynchopus* sp. YZ270 cl. 10.3, *Rhynchopus* sp. YZ270 cl. 9, *Rhynchopus* sp. DT0301, *Rhynchopus humris* YPF1505, *R. euleeides* ATCC 50226, *R. serpens* YPF1515, and *Sulcionema specki* YPF1618). *P. papillatum* and *R. euleeides* were isolated from coastal surface waters (United States) in 1985 and 1986, respectively. The remaining species originated either from coastal seawater around Japan or from Enoshima Aquarium (Kanagawa, Japan) and were continuously maintained in culture for 1 to 7 years prior to the analyses. The identity of not-yet-formally described species was established based on the 18S rRNA sequences as described previously (54). Dense cultures of trophic cells (55) were harvested by centrifugation at 3,000 × *g* for all subsequent analyses.

Light microscopy images and videos were taken with an Olympus BX53 microscope equipped with a DP72 microscope digital camera using CellSens software v. 1.11 (Olympus) and processed with GIMP v. 2.10.14, Irfan View v. 4.54, and Image J v. 1.51 software. Polarized microscopy was performed using crossed polarizers installed to a Raman microscope (as specified below).

### Environmental sampling.

Zooplankton was collected in the Bay of Villefranche sur Mer, France (43°40′N, 7°19′E) with a 10-min haul from 10 m to the surface, using a 20-μm-mesh-size plankton net. Captured copepods were transferred into 0.5 liters of freshly filtered natural seawater and starved for 12 h. *Centropages typicus*, *Temora longicornis*, and *Acartia* sp. were then picked under a dissection microscope. All experiments were carried out at cultivation temperature of the prey species of diplonemids (13°C for *Lacrimia* sp. YPF1808, room temperature for *N. karyoxenos*). Ten copepods were kept in 20 mL of diplonemid culture (10^5^ cells mL^−1^) for 5 days, after which their fecal pellets were collected under a dissection microscope and immediately analyzed by Raman microscopy (as specified below).

### Raman microscopy.

For the *in situ* determination of the chemical composition of intracellular structures, a confocal Raman microscope (alpha300 RSA; WITec, Germany) was used as previously described (56–60). To immobilize the fast-moving flagellates on the quartz slide, 5 μL of the cell pellet was mixed with 5 μL of 1% (wt/vol) solution of low-melting-point agarose (catalog number 6351.5; Carl Roth, Germany), immediately spread as a single-cell layer between a quartz slide and coverslip, and sealed with CoverGrip sealant (Biotium, USA). Two-dimensional Raman maps were obtained with laser excitation at 532 nm (20 mW power at the focal plane) and oil-immersion objective UPlanFLN 100×, numerical aperture (NA) 1.30, or water-immersion objective UPlanSApo 60×, NA 1.20 (Olympus, Japan). A scanning step size of 200 nm in both directions and an integration time of 100 ms per voxel were used. A minimum of 30 cells were measured for each strain. Raman chemical maps were constructed by multivariate decomposition of the baseline-corrected spectra into the spectra of pure chemical components by using Project Plus 5.1 software (WITec, Germany).

### TEM, TEM-ED, and TEM-EDX.

The protocol for the basic sample preparation of all kinds of electron microscopy approaches listed here is described in detail elsewhere (61). We used it with minor modifications, as stated below. Cell pellets were transferred to specimen carriers and immediately frozen in the presence of 20% (wt/vol) bovine serum albumin solution using a high-pressure freezer (Leica EM ICE, Leica Microsystems, Austria). Freeze substitution was performed in the presence of 2% osmium tetroxide diluted in 100% acetone at −90°C. After 96 h, specimens were warmed to −20°C at a step of 5°C/h. After another 24 h, the temperature was increased to 3°C (3°C/h). At room temperature, samples were washed in acetone and infiltrated with 25%, 50%, and 75% acetone/resin mixture for 1 h at each step. Finally, samples were infiltrated in 100% resin and polymerized at 60°C for 48 h. Semithin (250 nm) and ultrathin (70 nm) sections were cut using a diamond knife, placed on copper grids, and stained with uranyl acetate and lead citrate. TEM micrographs were taken with a Mega View III camera (SIS) using a JEOL 1010 TEM operating at an accelerating voltage of 80 kV.

For TEM-EDX, 10 μL of pelleted *L. lanifica* cells was spread over a holey carbon-coated copper grid, washed twice with 10 μL of distilled water in order to reduce the sea salts from the culture medium, and allowed to dry by evaporation at ambient temperature. Semithin sections of resin-infiltrated blocks of *N. karyoxenos* were prepared as stated above. For the identification of the crystalline phase, sections were studied by TEM on an FEI Tecnai 20 system (LaB6, 120 kV) equipped with an Olympus SIS charge-coupled-device camera Veleta (2,048 by 2,048 pixels) and an EDAX windowless EDX detector Apollo XLTW for elemental analysis. The diffraction data were collected by means of 3D electron diffraction (ED) (62). The data processing was carried out using PETS software (63). Structure solution and refinement were performed in the computing system Jana2006 (64).

### Cryo-scanning electron microscopy with EDX.

Cells pellets were high-pressure frozen as described above and transferred into a Leica ACE 600 preparation chamber (Leica Microsystems, Austria) precooled at −135°C, fractured with a scalpel, freeze-etched at −100°C for 1 min, and sputter-coated with 2.5 nm of gold-palladium at −125°C. Specimens were transferred under vacuum using transfer system VCT100 (Leica Microsystems, Austria) and observed with a Magellan 400L SEM (FEI, Czech Republic and USA) precooled at −125°C (cryo-SEM). Topographical images and EDX measurements were obtained using an EDT detector and EDAX detector (Octane Elect Super; EDAX, USA), respectively, either at 5 keV/0.1 NA or 10 keV/0.8 NA. The taken spectra were analyzed with EDAX TEAM software and quantified by the eZAF method.

### Serial block-face SEM.

The sample preparation of *Lacrimia* sp. YPF1808 by the high-pressure freezing technique followed the protocol for TEM sample preparation. After freeze-substitution, the samples were subsequently stained with 1% thiocarbohydrazide in 100% acetone for 1.5 h, 2% OsO_4_ in 100% acetone for 2 h at room temperature, and 1% uranyl acetate in 100% acetone overnight at 4°C. After every staining step, the samples were washed 3 times with 100% acetone for 15 min. Samples were then infiltrated with 25%, 50%, or 75% acetone-resin mixture for 2 h at each step, and finally infiltrated in 100% Hard Resin Plus 812 (EMS) overnight and polymerized at 62°C for 48 h. Resin-embedded blocks were trimmed and imaged using an Apreo SEM equipped with a VolumeScope (Thermo Fisher Scientific, Germany). Serial images were acquired at 3.5 keV, 50 pA, 40 Pa with a resolution of 6 nm, 100-nm slice thickness, and dwell time per pixel of 4 μs. Image data were processed in Microscopy Image Browser v2.702 (65) and Amira v2020.2. The resin-embedded blocks were also collected in the form of 1-μm-thick sections on a silicon wafer and analyzed by SEM-EDX (Magellan 400L system, as described above).

Based on volumetric data, we calculated the percentage of increase in cell density based on measured volumes of crystals compared to the theoretical crystal-free cells of the same volume and reported average theoretical density of 1.07 g·cm^−3^ (37).

### ICP-MS.

For analysis of Ba and ^88^Sr concentrations, cultures were grown in triplicates, counted, and washed three times with 1 M sorbitol solution (for *P. papillatum*, *D. japonicum*, and *Rhynchopus* YZ270 cl. 10) or Sr- and Ba-free artificial seawater rinsing solution (see above) (for *L. lanifica* JW1601, *Lacrimia* sp. 1808, and *N. karyoxenos*) to remove Ba^2+^ and Sr^2+^ present in the cultivation medium. Cultivated cells were harvested by centrifugation and rinsed twice with 50 mL and once with 2 mL of the rinsing solution, and the resulting pellets were freeze-dried. A 0.5-mL digestion acid mix (425 μL of 70% HClO_4_ and 75 μL of 69% HNO_3_) prepared as described elsewhere (66) was added directly to the dried biomass. The digestion was done using a Fuji PXG4 Thermoblock (AHF Analysentechnik AG, Germany). After evaporation of the acid mix, 0.5 mL of 5% HCl was added to each test tube to redissolve the salts. The glass tubes were heated to 90°C for 1 h to obtain clear solutions. The final volume of 1.5 mL was adjusted with double-distilled H_2_O. Appropriate dilutions were made with 0.2% HNO_3_. Indium was added as an internal standard at 1 ng/mL to each test solution. The ICP multielement standard solution VI (Merck, Germany) was used to prepare standard curves. Analyses were done using an inductively coupled plasma sector-field mass spectrometer (ICP sfMS) Element XR-2 with jet interface (Thermo Fisher Scientific, Germany) following a described protocol (67). Medium resolution of 4,000 was used in Ba and ^88^Sr measurements in triplicate of each technical replicate, with the highest precision and lowest relative standard deviation. Additionally, the elemental composition of samples of standard growth medium and artificial seawater medium without sulfates, Ba^2+^, or Sr^2+^ were analyzed.

### Holographic microscopy and QPI.

Samples for holographic microscopy were immobilized prior to measurement as described above for Raman microscopy. Imaging was performed at the Q-Phase microscope (Tescan Orsay Holding, Czech Republic). The holographic Q-Phase microscope is equipped with halogen lamp illumination through an interference filter (λ[¼] 650 nm, 10 nm full-width, half-maximal) and microscope objective (Nikon Plan Fluor oil immersion, 60×, numerical aperture 1.4, providing lateral resolution of 0.57 μm). The numerical reconstruction of acquired data was performed using Q-Phase software (Tescan Orsay Holding, Czech Republic). The technique enables automated cell segmentation and quantitative analysis of cellular mass based on the specific proportions of thickness and refractive indices of measured cells in comparison to the reference (68). Due to the high variability of cell contents and sizes, at least 150 cells were analyzed for each strain. Because crystalline inclusions caused artifacts during capturing due to the big difference in refractive indices, we analyzed crystal-free cells cultivated in the artificial seawater medium lacking Sr^2+^, Ba^2+^, and sulfates (as specified above). We calculated the total dry mass of the cells as the sum of crystal-free cells, measured by holographic microscopy, and SrSO_4_ and BaSO_4_ amounts measured via ICP-MS. The dry weight ratios of trace elements measured via ICP-MS were calculated based on the total dry weight of corresponding strains.

### Statistical data analysis.

Statistical analysis was conducted using SigmaPlot v. 12.5 and SPSS v. 23.0. Logarithmically normalized data were subjected to statistical tests (one-way analysis of variance [ANOVA] and Tukey’s *post hoc*) on an alpha level of 0.05. Calculations of standard errors of the means based on independent methods (i.e., ICP-MS quantification and QPI dry mass quantification) with different levels of variability were done according to mathematical conversion using Taylor expansion.

### Data availability.

All data generated or analyzed during this study are included in the published article and its supplemental material files.
