# Supplementary figures and images for: Massive Accumulation of Strontium and Barium in Diplonemid Protists
Source: mBio. 2023 Jan 16;14(1):e03279-22. doi: 10.1128/mbio.03279-22 (PMC9972996; doi:10.1128/mbio.03279-22)

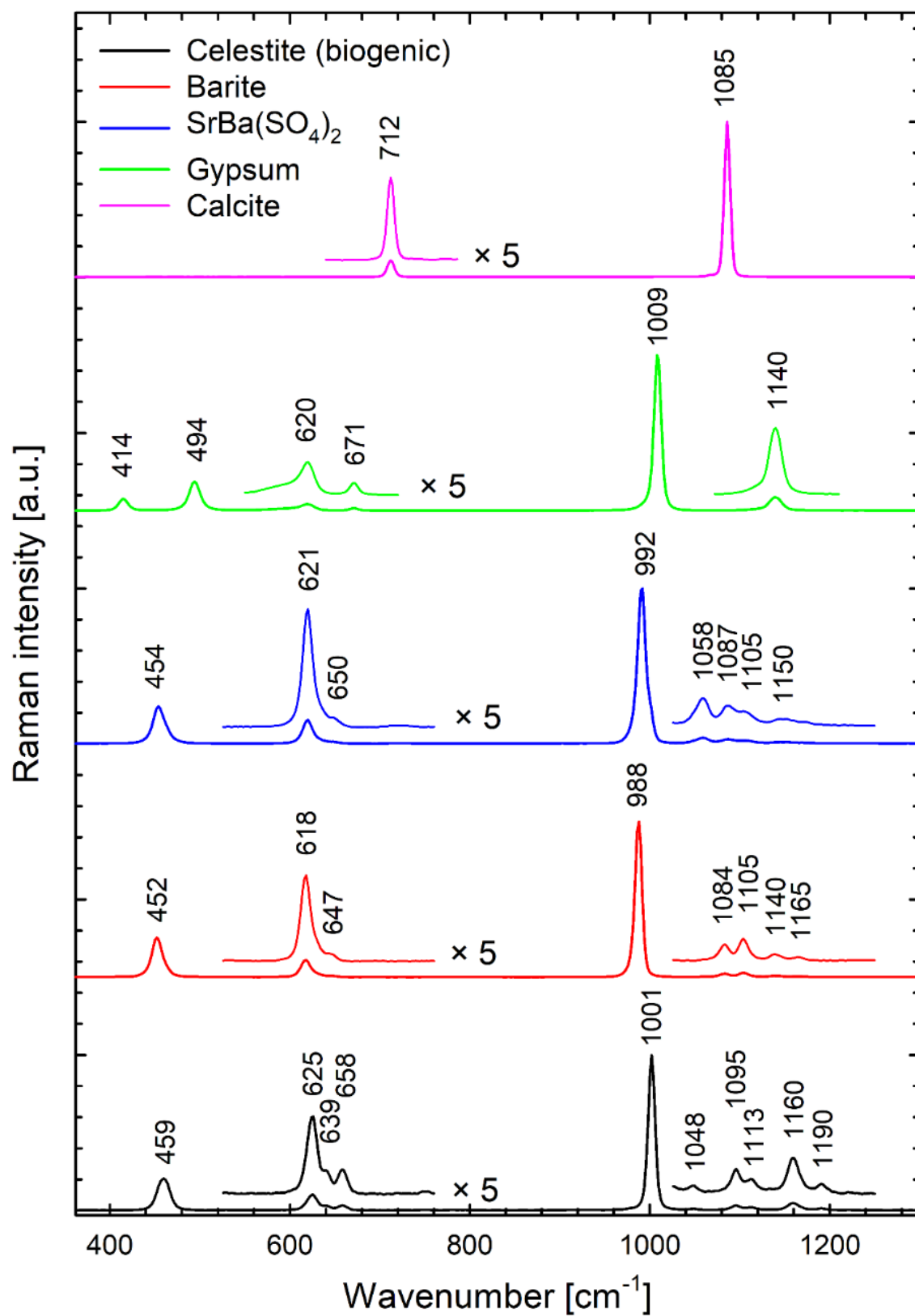

Supplement: FIG S1 [file mbio.03279-22-s0006.pdf]

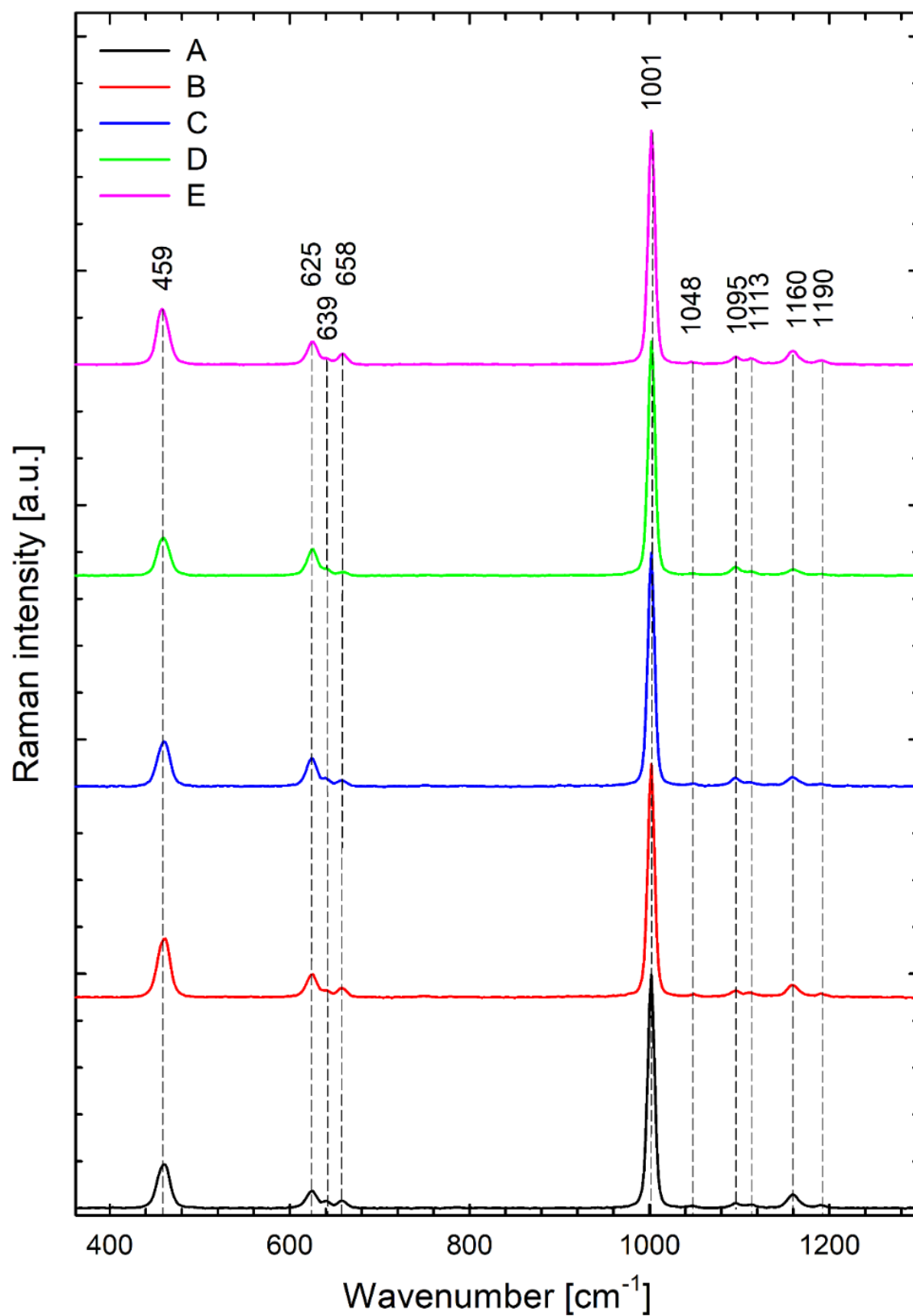

Supplement: FIG S2 [file mbio.03279-22-s0005.pdf]

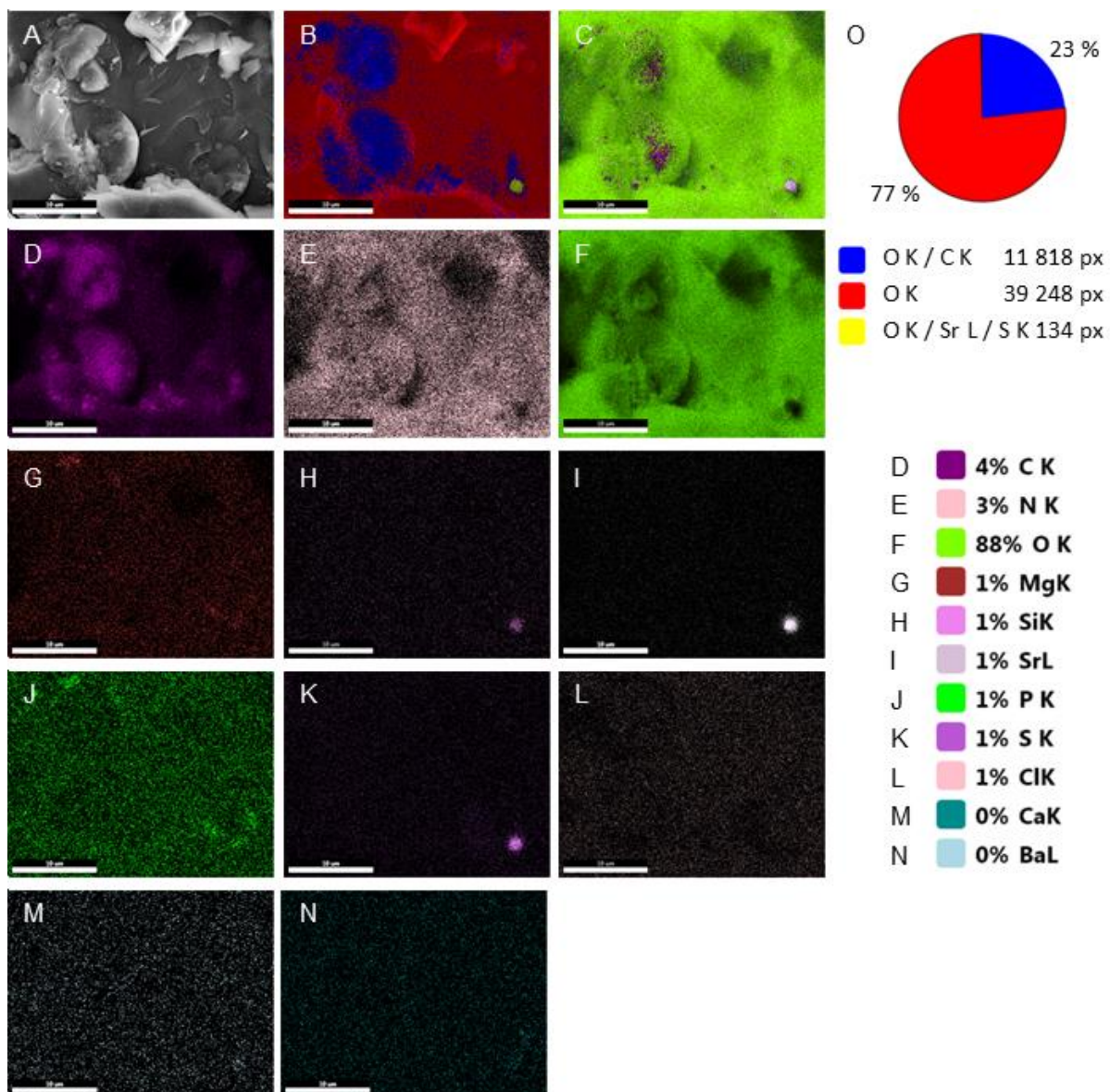

Supplement: FIG S3 [file mbio.03279-22-s0004.pdf]

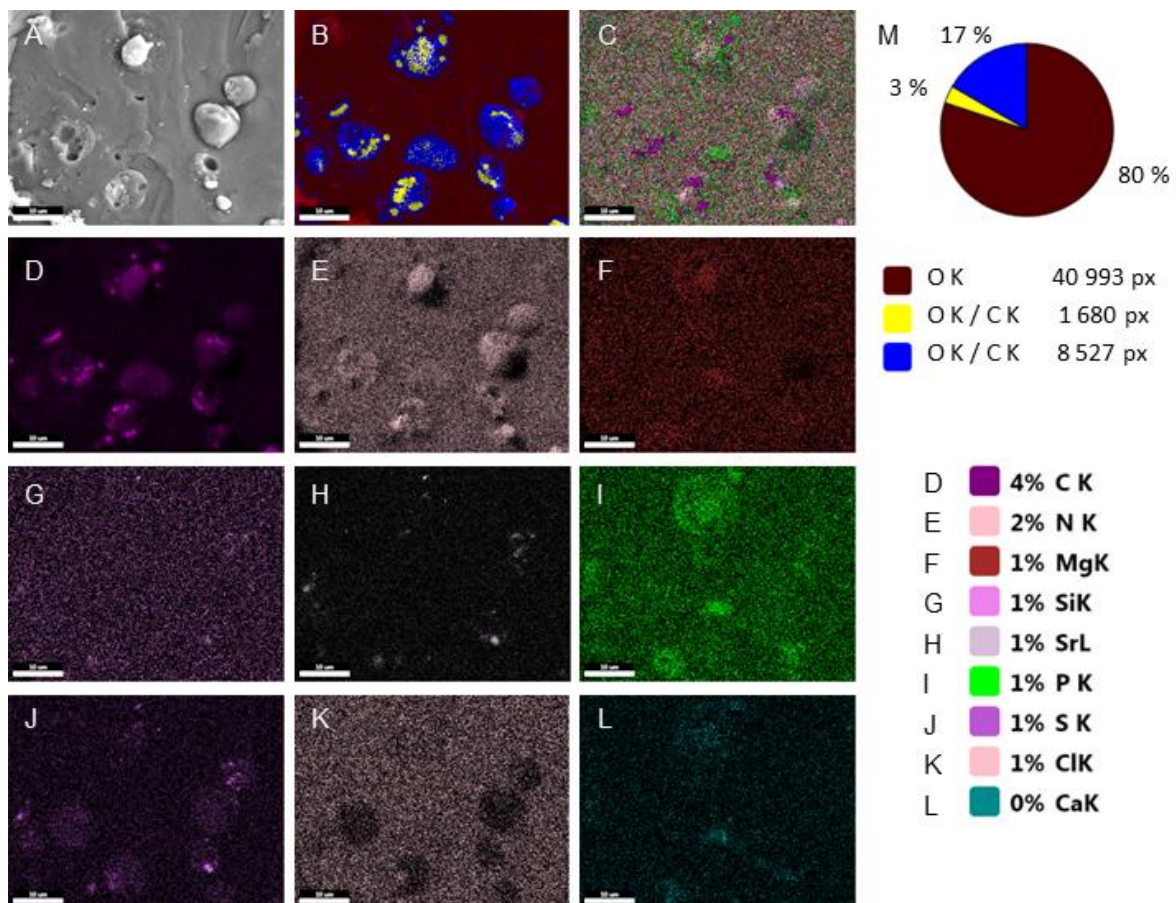

Supplement: FIG S4 [file mbio.03279-22-s0003.pdf]

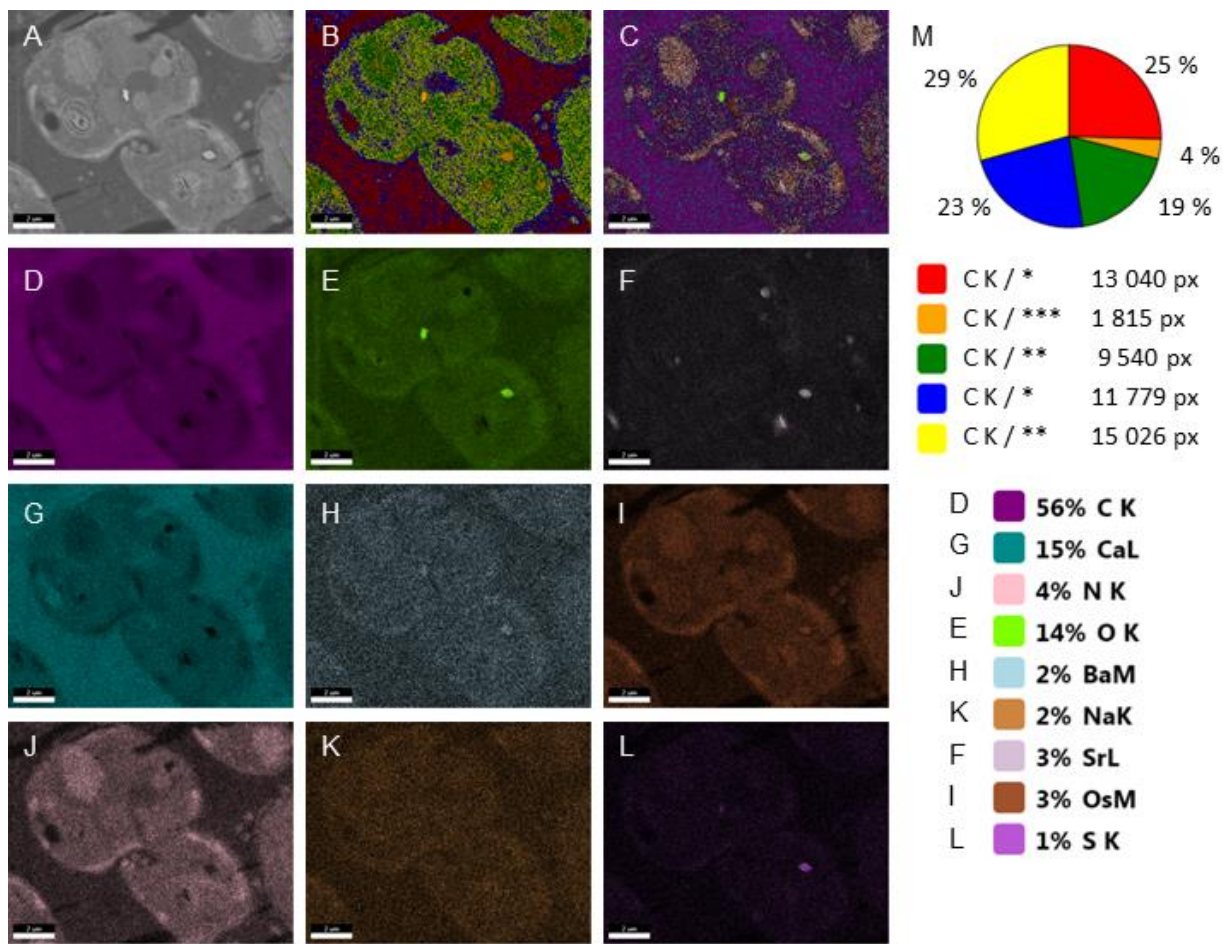

Supplement: FIG S5 [file mbio.03279-22-s0007.pdf]
