# Supplementary material for: Massive Accumulation of Strontium and Barium in Diplonemid Protists
Source: mBio. 2023 Jan 16;14(1):e03279-22. doi: 10.1128/mbio.03279-22 (PMC9972996; doi:10.1128/mbio.03279-22)
Supplement: TABLE S2 [file mbio.03279-22-s0001.pdf]

Table S2A. ICP-MS data and calculations - page 1/2

Sr amounts in 6 species Mean value Mean of 3 tech.repl

Species	Strain	Sample	Cell dry weight	Sr	Sr	Sr	$SrSO_4$	Sr	$SrSO_4$	Legend
			pg/cell	atoms/cell	fmol/cell	pg/cell	pg/cell	mg/g DW	mg/g DW	
Namystinia karyoxenos	YPF1621	x1	1421,1	2,88E+12	4790,15	419,713	879,855	295,35	619,14	"atoms/cell" = atoms Sr/Ba in sample/N of cells in sample
Namystinia karyoxenos	YPF1621	x2	1421,1	4,00E+12	6646,40	582,358	1220,811	409,80	859,07	"fmol/cell" = (atoms/cell / NA 6.022E23)*1E15
Namystinia karyoxenos	YPF1621	x3	1421,1	3,11E+12	5162,02	452,296	948,159	318,28	667,21	"pg/cell" = fmol/cell*Aw/1E3
Lacrimia sp.	YPF1808	x1	248,1	2,71E+11	450,28	39,454	82,708	159,01	333,33	"mg/g DW" = "pg/cell"/pg DW of 1 cell by holographic microscopy
Lacrimia sp.	YPF1808	x2	248,1	2,36E+11	391,45	34,299	71,901	138,23	289,78	"SrSO4 mg/g DW" = 1:1 stoichiometry of Sr:SO4
Lacrimia sp.	YPF1808	x3	248,1	1,54E+11	255,63	22,398	46,954	90,27	189,23	"BaSO4 mg/g DW" = 1:1 stoichiometry of Ba:SO4
Lacrimia lanifica	JW1601	x1	74,1	4,05E+10	67,24	5,891	12,350	79,50	166,66	"(Ba,Sr)SO4 mg/g DW" is a sum of BaSO4 and SrSO4
Lacrimia lanifica	JW1601	x2	74,1	3,45E+10	57,32	5,023	10,529	67,78	142,09	
Lacrimia lanifica	JW1601	x3	74,1	2,32E+10	38,60	3,382	7,090	45,64	95,68	**constants**
Rhynchopus sp. YZ270	cl. 10.3	x1	104,0	1,36E+07	0,02	0,002	0,004	0,02	0,04	NA - Avogadro's constant = 6.022E23
Rhynchopus sp. YZ270	cl. 10.3	x2	104,0	1,52E+07	0,03	0,002	0,005	0,02	0,04	Aw(Sr) = 87.62
Rhynchopus sp. YZ270	cl. 10.3	x3	104,0	1,29E+07	0,02	0,002	0,004	0,02	0,04	Aw(Ba) = 137.3
Diplonema japonicum	YPF1604	x1	109,7	1,18E+08	0,20	0,017	0,036	0,16	0,33	Mw(SO4) = 96.1
Diplonema japonicum	YPF1604	x2	109,7	1,35E+08	0,22	0,020	0,041	0,18	0,37	
Diplonema japonicum	YPF1604	x3	109,7	1,40E+08	0,23	0,020	0,043	0,19	0,39	
Diplonema papillatum	ATCC 50162	x1	68,0	7,55E+06	0,01	0,001	0,002	0,02	0,03	
Diplonema papillatum	ATCC 50162	x2	68,0	7,61E+06	0,01	0,001	0,002	0,02	0,03	
Diplonema papillatum	ATCC 50162	x3	68,0	8,48E+06	0,01	0,001	0,003	0,02	0,04	

Ba amounts in 6 species Mean of 3 tech.repl

Species	Strain	Sample	Cell dry weight	Ba	Ba	Ba	$BaSO_4$	Ba	$BaSO_4$	$(Ba,Sr)SO_4$
			pg/cell	atoms/cell	fmol/cell	pg/cell	pg/cell	mg/g DW	mg/g DW	mg/g DW
Namystinia karyoxenos	YPF1621	x1	1421,1	6,26E+11	1039,36	142,73	242,574	100,44	170,70	789,84
Namystinia karyoxenos	YPF1621	x2	1421,1	8,80E+11	1460,48	200,56	340,857	141,13	239,86	1098,93
Namystinia karyoxenos	YPF1621	x3	1421,1	6,67E+11	1108,37	152,21	258,678	107,11	182,03	849,24
Lacrimia sp.	YPF1808	x1	248,1	4,74E+10	78,76	10,82	18,381	43,59	74,08	407,41
Lacrimia sp.	YPF1808	x2	248,1	4,24E+10	70,44	9,67	16,441	38,99	66,26	356,04
Lacrimia sp.	YPF1808	x3	248,1	2,72E+10	45,09	6,19	10,523	24,95	42,41	231,64
Lacrimia lanifica	JW1601	x1	74,1	1,21E+09	2,01	0,28	0,468	3,72	6,32	172,98
Lacrimia lanifica	JW1601	x2	74,1	1,72E+09	2,86	0,39	0,667	5,30	9,00	151,09
Lacrimia lanifica	JW1601	x3	74,1	7,08E+08	1,18	0,16	0,274	2,18	3,70	99,38
Rhynchopus sp. YZ270	cl. 10.3	x1	104,0	N/A	N/A	N/A	N/A	N/A	N/A	N/A
Rhynchopus sp. YZ270	cl. 10.3	x2	104,0	N/A	N/A	N/A	N/A	N/A	N/A	N/A
Rhynchopus sp. YZ270	cl. 10.3	x3	104,0	N/A	N/A	N/A	N/A	N/A	N/A	N/A
Diplonema japonicum	YPF1604	x1	109,7	N/A	N/A	N/A	N/A	N/A	N/A	N/A
Diplonema japonicum	YPF1604	x2	109,7	N/A	N/A	N/A	N/A	N/A	N/A	N/A
Diplonema japonicum	YPF1604	x3	109,7	N/A	N/A	N/A	N/A	N/A	N/A	N/A
Diplonema papillatum	ATCC 50162	x1	68,0	N/A	N/A	N/A	N/A	N/A	N/A	N/A
Diplonema papillatum	ATCC 50162	x2	68,0	N/A	N/A	N/A	N/A	N/A	N/A	N/A
Diplonema papillatum	ATCC 50162	x3	68,0	N/A	N/A	N/A	N/A	N/A	N/A	N/A

Table S2A. ICP-MS data and calculations - page 2/2

Cell counts, volume of samples

Sorbitol as rinsing solution

Strain	Replicate	Cells/ml	Culture volume	Total cells
Rhynchopus YZ270 cl. 10.3*	x1	9,09E+05	45	4,09E+07
Rhynchopus YZ270 cl. 10.3*	x2	9,13E+05	45	4,11E+07
Rhynchopus YZ270 cl. 10.3*	x3	1,02E+06	45	4,57E+07
Diplonema japonicum YPF1604*	x1	1,93E+05	220	4,25E+07
Diplonema japonicum YPF1604*	x2	2,09E+05	225	4,70E+07
Diplonema japonicum YPF1604*	x3	2,25E+05	185	4,16E+07
Diplonema papillatum ATCC 501	x1	2,64E+06	50	1,32E+08
Diplonema papillatum ATCC 501	x2	2,03E+06	50	1,02E+08
Diplonema papillatum ATCC 501	x3	1,99E+06	50	9,95E+07

Cell counts, volume of samples, theoretical depletion ratio

Sr- and Ba-free artificial seawater as rinsing solution

Strain	Replicate	Cells/ml	Sample volume	Total cells	Initial volume of culture, ml
Lacrimia sp. YPF1808*	x1	3,59E+06	3,95	1,42E+07	250
Lacrimia sp. YPF1808*	x2	3,49E+06	4,15	1,45E+07	250
Lacrimia sp. YPF1808*	x3	4,03E+06	3,95	1,59E+07	250
Lacrimia lanifica JW1601**	x1	7,35E+05	247	1,82E+08	247
Lacrimia lanifica JW1601**	x2	8,05E+05	245	1,97E+08	245
Lacrimia lanifica JW1601**	x3	7,25E+05	244	1,77E+08	244
Namystinia karyoxenos YPF1621	x1	1,37E+06	2,6	3,56E+06	100
Namystinia karyoxenos YPF1621	x2	1,21E+06	2,2	2,66E+06	100
Namystinia karyoxenos YPF1621	x3	1,40E+06	3,22	4,51E+06	100

*Counted after 2 round of centrifugation

**Counted directly from cultures, before centrifugation

Table S2B. Concentration of Ba and Sr inside the cells relative to the concentration in the surrounding medium - page 1/2

			Ba			Sr		
**Species**	**Strain**	**Replicate**	**Atoms/cell measured by ICP-MS**	**Atoms/cell calculated* theoretical value**	**Measured/theoretical, factor**	**Atoms/cell measured by ICP-MS**	**Atoms/cell calculated* theoretical value**	**Measured/theoretical, factor**
Namystinia karyoxenos	YPF1621	x1	6,26E+11	2,10E+07	29864,21	2,88E+12	4,10E+08	7037,41
Namystinia karyoxenos	YPF1621	x2	8,80E+11	2,21E+07	39715,41	4,00E+12	4,33E+08	9241,21
Namystinia karyoxenos	YPF1621	x3	6,67E+11	1,17E+07	57055,80	3,11E+12	2,29E+08	13586,74
**Average**			**7,24E+11**	**1,83E+07**	**42211,81**	**3,33E+12**	**3,57E+08**	**9955,12**
Lacrimia sp.	YPF1808	x1	4,74E+10	8,01E+06	5918,10	2,71E+11	1,57E+08	1729,98
Lacrimia sp.	YPF1808	x2	4,24E+10	8,96E+06	4734,67	2,36E+11	1,75E+08	1345,24
Lacrimia sp.	YPF1808	x3	2,72E+10	1,21E+07	2242,96	1,54E+11	2,37E+08	650,22
**Average**			**3,90E+10**	**9,69E+06**	**4298,58**	**2,20E+11**	**1,90E+08**	**1241,81**
Lacrimia lanifica	JW1601	x1	1,21E+09	1,02E+06	1179,25	4,05E+10	2,00E+07	2019,98
Lacrimia lanifica	JW1601	x2	1,72E+09	9,81E+05	1755,04	3,45E+10	1,92E+07	1800,01
Lacrimia lanifica	JW1601	x3	7,08E+08	1,58E+06	448,64	2,32E+10	3,09E+07	753,29
**Average**			**1,21E+09**	**1,19E+06**	**1127,64**	**3,28E+10**	**2,34E+07**	**1524,43**

* - an estimation on how many atoms a cell would contain if it was in equilibrium with the surrounding medium, calculated as shown below (column H)

**Species**	**Strain**	**Replicate**	**Atoms/1 mg of Hemi medium**	**Weight of pellet, mg**	**Atoms per theoretical pellet**	**N of cells in actual pellet**	**Atoms per theoretical cell**	
Namystinia karyoxenos	YPF1621	x1	2,80E+12	26,67	7,47E+13	3,56E+06	**2,10E+07**	**Ba**
Namystinia karyoxenos	YPF1621	x2	2,80E+12	21,06	5,90E+13	2,66E+06	**2,21E+07**	
Namystinia karyoxenos	YPF1621	x3	2,80E+12	18,84	5,27E+13	4,51E+06	**1,17E+07**	
Lacrimia sp.	YPF1808	x1	2,80E+12	40,6	1,14E+14	1,42E+07	**8,01E+06**	
Lacrimia sp.	YPF1808	x2	2,80E+12	46,36	1,30E+14	1,45E+07	**8,96E+06**	
Lacrimia sp.	YPF1808	x3	2,80E+12	68,84	1,93E+14	1,59E+07	**1,21E+07**	
Lacrimia lanifica	JW1601	x1	2,80E+12	66,47	1,86E+14	1,82E+08	**1,02E+06**	
Lacrimia lanifica	JW1601	x2	2,80E+12	69,09	1,93E+14	1,97E+08	**9,81E+05**	
Lacrimia lanifica	JW1601	x3	2,80E+12	99,71	2,79E+14	1,77E+08	**1,58E+06**	
Namystinia karyoxenos	YPF1621	x1	5,47E+13	26,67	1,46E+15	3,56E+06	**4,10E+08**	**Sr**
Namystinia karyoxenos	YPF1621	x2	5,47E+13	21,06	1,15E+15	2,66E+06	**4,33E+08**	
Namystinia karyoxenos	YPF1621	x3	5,47E+13	18,84	1,03E+15	4,51E+06	**2,29E+08**	
Lacrimia sp.	YPF1808	x1	5,47E+13	40,6	2,22E+15	1,42E+07	**1,57E+08**	
Lacrimia sp.	YPF1808	x2	5,47E+13	46,36	2,54E+15	1,45E+07	**1,75E+08**	
Lacrimia sp.	YPF1808	x3	5,47E+13	68,84	3,77E+15	1,59E+07	**2,37E+08**	
Lacrimia lanifica	JW1601	x1	5,47E+13	66,47	3,64E+15	1,82E+08	**2,00E+07**	
Lacrimia lanifica	JW1601	x2	5,47E+13	69,09	3,78E+15	1,97E+08	**1,92E+07**	
Lacrimia lanifica	JW1601	x3	5,47E+13	99,71	5,46E+15	1,77E+08	**3,09E+07**	

Table S2B. Concentration of Ba and Sr inside the cells relative to the concentration in the surrounding medium - page 2/2

Legend

Column	Meaning
"D"	Number of atoms in 1 µl of Hemi medium based on ICP-MS measurements of the medium (calculated below)
"E"	Wet weight of cell pellets (rinsed with Ba/Sr-free medium), which was provided for ICP-MS measurements
"F"	D*E - total number of atoms that each pellet would contain if cells were in equilibrium with the medium and did not concentrate Ba/Sr
"G"	Number of cells in pellets provided for the ICP-MS measurements
"H"	E/F - number of atoms a cell would contain if it was in equilibrium with the medium and did not concentrate Ba/Sr

Calculation of number of atoms are in 1 ul of Hemi medium based on ICP-MS measurements of the medium

	MW g/mol	Concentration* g/l	Concentration mol/l	Concentration atoms/l	Atoms/µl or mg of Hemi medium**
Sr	88	0,008	9,09E-05	5,47E+19	5,47E+13
Ba	137,33	0,00064	4,65E-06	2,80E+18	2,80E+12

* - resuts of ICP-MS measurement
** - not considering difference in density (1.03g/ml)

Table S2C. Volumetric measurements of diplonemid Lacrimia sp. YPF1808 based on SBF-SEM 3D reconstruction - page 1/3

Volumetric measurements of diplonemid *Lacrimia* sp. YPF1808 based on SBF-

**Cell**	Vol. cryst 1 (µm^3)	Vol. cryst 2 (µm^3)	Vol. cryst 3 (µm^3)	Vol. cryst 4 (µm^3)	Vol. cryst 5 (µm^3)	Vol. cryst 6 (µm^3)	Vol. cryst 7 (µm^3)	Vol. cryst 8 (µm^3)	Vol. cryst 9 (µm^3)	Vol. cryst 10 (µm^3)	Vol. Cryst 11 (µm^3)	Vol. cryst 12 (µm^3)	Vol. cryst 13 (µm^3)	Vol. cryst 14 (µm^3)	Vol. cryst 15 (µm^3)	Vol. cryst 16 (µm^3)
1	2,704	0,942	-	-	-	-	-	-	-	-	-	-	-	-	-	-
2	0,769	0,214	0,052	-	-	-	-	-	-	-	-	-	-	-	-	-
3	1,708	0,523	0,500	-	-	-	-	-	-	-	-	-	-	-	-	-
4	0,962	0,027	0,011	0,009	-	-	-	-	-	-	-	-	-	-	-	-
5	0,037	0,017	-	-	-	-	-	-	-	-	-	-	-	-	-	-
6	0,170	0,130	0,124	0,116	0,061	0,054	0,026	0,015	-	-	-	-	-	-	-	-
7	0,208	0,013	0,009	0,009	0,008	0,007	0,007	0,007	0,007	0,007	0,005	0,005	0,005	0,005	0,003	0,002
8	0,115	0,101	0,029	0,009	-	-	-	-	-	-	-	-	-	-	-	-
9	0,150	0,010	-	-	-	-	-	-	-	-	-	-	-	-	-	-
10	0,211	0,195	0,004	-	-	-	-	-	-	-	-	-	-	-	-	-
11	0,433	0,323	0,109	-	-	-	-	-	-	-	-	-	-	-	-	-
12	0,383	0,067	0,056	0,030	0,028	0,027	0,014	0,013	0,012	0,010	0,009	-	-	-	-	-
13	0,274	0,192	0,012	0,011	0,008	0,004	-	-	-	-	-	-	-	-	-	-
14	0,089	0,053	0,038	-	-	-	-	-	-	-	-	-	-	-	-	-
15	1,080	0,264	0,072	0,034	0,012	0,007	0,003	-	-	-	-	-	-	-	-	-
16	1,353	0,503	0,436	0,106	0,014	-	-	-	-	-	-	-	-	-	-	-
17	2,664	0,045	-	-	-	-	-	-	-	-	-	-	-	-	-	-
18	0,140	0,125	0,119	0,100	0,072	0,023	0,008	0,006	0,005	0,003	-	-	-	-	-	-
19	0,084	0,056	0,016	0,003	-	-	-	-	-	-	-	-	-	-	-	-
20	0,171	0,124	0,089	0,087	0,013	0,004	0,003	-	-	-	-	-	-	-	-	-
21	6,968	-	-	-	-	-	-	-	-	-	-	-	-	-	-	-

**Cell**	**Number of crystals**	**Total volume of crystals (µm^3)**	**Average size of 1 crystal (µm^3)**	**Volume of the cell (µm^3)**	**Ratio of cell volume / crystal**	**Ratio of crystal volume / cell**	**Volumetric percentage of crystal (%)**
1	2	**3,646**	1,823	148,930	40,849	0,024	2,448
2	3	**1,036**	0,345	175,930	169,885	0,006	0,589
3	3	**2,732**	0,911	251,160	91,937	0,011	1,088
4	4	**1,008**	0,252	315,810	313,191	0,003	0,319
5	2	**0,054**	0,027	328,390	6092,262	0,000	0,016
6	8	**0,696**	0,087	404,150	580,337	0,002	0,172
7	16	**0,306**	0,019	411,010	1345,180	0,001	0,074
8	4	**0,254**	0,064	470,300	1848,341	0,001	0,054
9	2	**0,160**	0,080	489,070	3059,479	0,000	0,033
10	3	**0,410**	0,137	502,410	1226,509	0,001	0,082
11	3	**0,864**	0,288	518,000	599,631	0,002	0,167
12	11	**0,649**	0,059	626,010	965,266	0,001	0,104
13	6	**0,500**	0,083	660,190	1319,325	0,001	0,076
14	3	**0,180**	0,060	789,630	4390,609	0,000	0,023
15	7	**1,473**	0,210	947,900	643,551	0,002	0,155
16	5	**2,412**	0,482	975,460	404,414	0,002	0,247
17	2	**2,710**	1,355	987,680	364,503	0,003	0,274
18	10	**0,601**	0,060	1131,700	1884,546	0,001	0,053
19	4	**0,160**	0,040	1134,900	7114,807	0,000	0,014
20	7	**0,491**	0,070	1136,000	2315,494	0,000	0,043
21	1	**6,968**	6,968	620,200	89,002	0,011	1,124

Table S2C. Volumetric measurements of diplonemid Lacrimia sp. YPF1808 based on SBF-SEM 3D reconstruction - page 2/3

Theoretical cell density based on literature

Density	value	unit
**ρ (cell min)**	**0,985**	g/cm^3
**ρ (cell max)**	**1,156**	g/cm^3
**ρ (cell mean)**	**1,0705**	g/cm^3
**ρ ($SrSO_4$)**	**3,9**	g/cm^3

REFERENCE: Cellular density range based on the reference: Walsby, A. E. & Reynolds, C. S. Sinking and floating. in The Physiological Ecology of the Phytoplankton. (ed. Morris, I.) 371–412 (Blackwell Science, 1980).

Calculations of cell and crystal densities

Cell	Volume of cell	Volume of crystals (cumulative)	Density of cells with crystals	Increase in cell density
	μm^3	μm^3	g/cm^3	%
1	148,93	3,65	1,14	6,32
2	175,93	1,04	1,09	1,55
3	251,16	2,73	1,10	2,84
4	315,81	1,01	1,08	0,84
5	328,39	0,05	1,07	0,04
6	404,15	0,70	1,08	0,45
7	411,01	0,31	1,07	0,20
8	470,3	0,25	1,07	0,14
9	489,07	0,16	1,07	0,09
10	502,41	0,41	1,07	0,22
11	518	0,86	1,08	0,44
12	626,01	0,65	1,07	0,27
13	660,19	0,50	1,07	0,20
14	789,63	0,18	1,07	0,06
15	947,9	1,47	1,07	0,41
16	975,46	2,41	1,08	0,65
17	987,68	2,71	1,08	0,72
18	1131,7	0,60	1,07	0,14
19	1134,9	0,16	1,07	0,04
20	1136	0,49	1,07	0,11
21	620,2	6,97	1,10	2,94
**Mean**	**620,23**	**1,30**	**1,08**	**0,89**
SEM	70,36	0,36	0,00	0,33

Legend

Based on measured cell and crystal volumes via SBF-SEM and the average theoretical cell density 1,0705 g/cm^3 and celestite density 3,9 g/cm^3, the increase in the overall cell density with crystals was counted
* crystal volumes are less than 1 % of cell volume, therefore we neglected their volumes in the overall cellular volume

Table S2C. Volumetric measurements of diplonemid Lacrimia sp. YPF1808 based on SBF-SEM 3D reconstruction - page 3/3

Theoretical sedimentation rates assumed based on Stokes' law:

Physical property	value	unit
g	9,81	m/s^2
R	0,00001	m
ρ_p **1***	900	kg/m^3
ρ_p **2**	1050	kg/m^3
ρ_p **3****	1100	kg/m^3
ρ_p **4**	1200	kg/m^3
ρ_p **5*****	1300	kg/m^3
ρ_f	1030	kg/m^3
μ	0,00089	kg/m/s

Legend

* Negative buyoncy

** Values that could be reached in case of *Lacrimia* sp. YPF 1808 based on 3D reconstructions of SBF-SEM

*** Predicted values for *Namystinia karyoxenos* based on extrapolation of ICP MS quantifications on 3D reconstructions in Lacrimia sp. YPF1808

$$v = \frac{2}{9}\frac{(\rho_p - \rho_f)}{\mu} g R^2$$

(vertically downwards if $\rho_p > \rho_f$, upwards if $\rho_p < \rho_f$), where:

- g is the gravitational field strength (m/s^2)
- R is the radius of the spherical particle (m)
- ρ_p is the mass density of the particle (kg/m^3)
- ρ_f is the mass density of the fluid (kg/m^3)
- μ is the dynamic viscosity (kg/(m*s)).

Potential particle/cell densities			Velocity	value	unit	value	unit	value	unit	value	unit	value	unit
ρ_p **1 ***	900	kg/m^3	*v1*	-3,18E-05	m/s	-31,84	µm/s	-2,75	m/day	-83	m/month	**-990**	**m/year**
ρ_p **2**	1050	kg/m^3	*v2*	4,9E-06	m/s	4,90	µm/s	0,42	m/day	13	m/month	**152**	**m/year**
ρ_p **3 ****	1100	kg/m^3	*v3*	1,71E-05	m/s	17,15	µm/s	1,48	m/day	44	m/month	**533**	**m/year**
ρ_p **4**	1200	kg/m^3	*v4*	4,16E-05	m/s	41,64	um/s	3,60	m/day	108	m/month	**1295**	**m/year**
ρ_p **5 *****	1300	kg/m^3	*v5*	6,61E-05	m/s	66,13	µm/s	5,71	m/day	171	m/month	**2057**	**m/year**
