# Supplementary material for: Massive Accumulation of Strontium and Barium in Diplonemid Protists
Source: mBio. 2023 Jan 16;14(1):e03279-22. doi: 10.1128/mbio.03279-22 (PMC9972996; doi:10.1128/mbio.03279-22)
Supplement: TABLE S1 [file mbio.03279-22-s0002.pdf]

		Standard seawater growth medium		Artificial seawater medium without the addition of sulfates, Ba and Sr	
Element	unit	mean	SD	mean	SD
Mg	ppb	1 710 000	69 000	3 190 000	120 000
	nM	71 200 000	2 900 000	132 900 000	4 900 000
K	ppb	1 360 000	190 000	1 291 000	52 000
	nM	34 800 000	4 900 000	33 100 000	1 300 000
Ca	ppb	513 000	34 000	545 000	18 000
	nM	11 650 000	770 000	12 390 000	420 000
S	ppb	165 000	12 000	2 570	120
	nM	1 870 000	140 000	29 200	1 300
^{56}Fe	ppb	500	240	1 170	140
	nM	9 000	4 400	20 800	2 400
P	ppb	1 100	1 300	630	630
	nM	35 000	42 000	20 000	20 000
Zn	ppb	320	70	303	27
	nM	4 900	1 100	4 600	400
^{88}Sr	ppb	7 870	440	246	16
	nM	89 400	5 000	2 800	180
Ba	ppb	638	21	291	17
	nM	4 590	150	2 100	120
Ni	ppb	101	4	95	4
	nM	1 680	73	1 585	61
Mn	ppb	46	2	79	8
	nM	832	31	1 430	140
Cr	ppb	50	3	49	4
	nM	956	66	948	76
Cu	ppb	88	58	55	10
	nM	1 390	930	870	160
